# Histological Image Processing Features Induce a Quantitative Characterization of Chronic Tumor Hypoxia

**DOI:** 10.1371/journal.pone.0153623

**Published:** 2016-04-19

**Authors:** Andrew Sundstrom, Elda Grabocka, Dafna Bar-Sagi, Bud Mishra

**Affiliations:** 1 Department of Pharmacology and Systems Therapeutics, Icahn School of Medicine at Mount Sinai, New York, NY, United States of America; 2 Department of Biochemistry and Molecular Pharmacology, NYU School of Medicine, New York, NY, United States of America; 3 Department of Computer Science, Courant Institute of Mathematical Sciences, New York, NY, United States of America; Universidade de São Paulo, BRAZIL

## Abstract

Hypoxia in tumors signifies resistance to therapy. Despite a wealth of tumor histology data, including anti-pimonidazole staining, no current methods use these data to induce a quantitative characterization of chronic tumor hypoxia in time and space. We use image-processing algorithms to develop a set of candidate image features that can formulate just such a quantitative description of xenographed colorectal chronic tumor hypoxia. Two features in particular give low-variance measures of chronic hypoxia near a vessel: intensity sampling that extends radially away from approximated blood vessel centroids, and multithresholding to segment tumor tissue into normal, hypoxic, and necrotic regions. From these features we derive a spatiotemporal logical expression whose truth value depends on its predicate clauses that are grounded in this histological evidence. As an alternative to the spatiotemporal logical formulation, we also propose a way to formulate a linear regression function that uses all of the image features to learn what chronic hypoxia looks like, and then gives a quantitative similarity score once it is trained on a set of histology images.

## Introduction

As a tumor grows, it rapidly outstrips its blood supply. High proliferation causes high cell density that overtaxes local oxygen supply. This leaves portions of the tumor with an oxygen concentration significantly lower than in healthy tissues. This stress condition is tumor hypoxia. Hypoxia is strongly correlated with poor prognosis as it renders tumors less responsive to chemotherapy and radiotherapy [1–3].

Hypoxia-inducible factors (HIFs) are transcription factors that respond to changes in available oxygen in the cellular environment, specifically to hypoxia. When activated, HIF-1 upregulates several genes to promote survival in low-oxygen conditions. These include glycolysis enzymes that allow cells to synthesize ATP in an oxygen-independent manner, and vascular endothelial growth factor (VEGF) that cells release to promote angiogenesis. So hypoxia is directly instrumental in tumor progression.

Prolonged or extreme hypoxia can lead to necrosis, and tumors often have central regions called necrotic cores [4]. Necrosis in turn activates inflammatory responses that produce cytokines that stimulate tumor growth [3]. Recent research has investigated the interactions between hypoxic tumor cells and immune cells (tumor-associated macrophages [5]) and cells that synthesize extracellular matrix (tumor-associated fibroblasts [6, 7]). Both are involved with inflammatory processes tied to tumor progression. In the context of the tumor microenvironment, these interactions regulate tumor properties like spatial patterns of cell localization, angiogenesis, and collective invasion and migration [8, 9].

Thus it is of theoretical and clinical significance to understand how, and under what conditions, hypoxia arises in tumors. More fundamentally, we need to better characterize tumor hypoxia from available evidence, so it can be reliably detected in its various states and contexts.

Tumor hypoxia exhibits two major forms—intermittent and chronic. *Intermittent* hypoxia derives from the pervasive presence of fluctuating oxygenation in whole tumors, and operates in a length scale that exceeds the locality of specific vessels [10]. *Chronic* hypoxia derives from a vessel-dominant oxygenation dynamics whose parameters correspond to vessel and tissue properties, and the radial distance from the vessel.

In this study we choose to investigate chronic tumor hypoxia situations where there is a presumed steady-state gradient of oxygen near a source vessel, diminishing in magnitude as a function of radial distance away from that vessel. This phenomenon has been investigated since the clinical study of Thomlinson and Gray [4] first characterized “tumor cords”. Red blood cells release oxygen by diffusion into the tumor tissue regions in need. The oxygen is metabolized by respiring cells near the blood vessel, and consequently oxygen tension diminishes as a radial distance away from the vessel. At radial distances in excess of ∼ 100 *μm*, there is insufficient oxygen to maintain cell viability. Between the bands of viable and necrotic cells, one typically finds a region 1–2 cell layers thick where oxygen tension is hypoxic—this is consistent with our tumor image data, described below. Moreover, in a solid tumor mass, mitotic index and cell viability decreases as a function of radial distance away from the nearest blood vessel [4, 11].

We develop a method to induce a quantitative characterization of these chronic tumor hypoxia situations from histological evidence, namely image data taken from H&E and anti-pimonidazole stained slices of tumors. In this way, we take a reductionist approach, which we understand does not integrate the full complexity of tumor vascularity and hypoxia. Rather, we choose to focus on our simplified biological system to better characterize it by way of our computational modeling techniques—assembling a logical description comprising quantitative image features, and assembling a linear function whose terms comprise quantitative image features and are weighted according to a linear regression. We show how one can use the image features we develop (and an unbounded set of other image features) to produce an automated, scalable, and unbiased spatiotemporal characterization of chronic tumor hypoxia.

The quantitative study of chronic hypoxia near blood vessels is part of a well established literature that seeks to improve our understanding of microvascular oxygen transport in tissues by building theoretical models.

Krogh (1919) was one of the first to systematically investigate the architectural relationship between blood capillaries and muscle cells, and the conditions under which oxygen flows from blood cells to muscle cells [12–14]. In particular, the Krogh cylinder model [13] gives a quantitative, predictive description of oxygen tension within an idealized system of a single capillary. It defines two concentric cylinders, one of muscle tissue (having radius *R*) surrounding another of vessel (having radius *r*); it describes the oxygen tension at distance *x* into the muscle tissue (*T*_*x*_) as a function of: the oxygen tension in the capillary (*pO*_2_), the diffusion constant for oxygen in muscle [12], the rate of oxygen consumption, and the the radii *R* and *r*. When *x* = *R*, the model gives the maximum tension difference (*T*_0_ − *T*_*R*_) necessary to supply the muscle with oxygen at any point along the capillary. If *T*_0_ − *T*_*R*_ is greater than the oxygen tension of venous blood, then the same portion of muscle (near the venous end of the capillaries) will be hypoxic; if *T*_0_ − *T*_*R*_ is less than the oxygen tension of venous blood, then oxygen tension is positive everywhere within the muscle tissue.

Krogh approached the complex problem of microvascular oxygen transport in tissues by parsing it into three aspects: “(1) The physical problem of the rate at which oxygen diffuses into and through the tissues; (2) the anatomical problem of the number and distribution of capillaries with respect to the cells; and (3) the physiological problem of regulating the supply of blood and by that the availability of oxygen under the conditions of rest and in exercise.” [15]. Since Krogh’s groundbreaking work, many researchers have developed theoretical models to address the biochemical, structural, geometric, and hemodynamic complexity involved in the problem. In the past two decades, multi-vessel models have been developed to consider microvascular arrays and networks. These models have shown the physiological significance of heterogeneities in vessel spacing, oxygen supply, flow path of red blood cells, and interactions between capillaries and arterioles [16].

Some of these models consider tumor tissue in various respects. Kang, *et al* [17] models oxygen transport during tumor hyperthermia. Kavanagh, *et al* [18] models tumor oxygenation under varying hemoglobin-oxygen affinities. Kirkpatrick, *et al* [19] explores the influence of kinetic and physical factors on substrate metabolism in a Krogh tumor model. Secomb, *et al* [20] presents theoretical simulations of oxygen delivery to tumor tissues by networks of microvessels, based on *in vivo* observations of vascular geometry and blood flow in the microcirculation in mammary adenocarcinoma tumors. This is a rich area of active research.

In our study, we are not concerned with modeling any particular aspect of microvascular oxygen transport in tissues, let alone the full dimensionality of this complex phenomenon. While we acknowledge the Krogh and multi-vessel models could provide their own set of quantitative features for characterizing chronic tumor hypoxia near a vessel, we have taken a simpler approach, to empirically measure the anti-pimonidazole gradients by image analysis. Such measured gradients are but one of a potentially large set of image features to be combined logically and functionally in a later phase of processing, discussed below.

Helmlinger, *et al* [21] experimentally measured interstitial *pO*_2_ *in vivo* in a number of xenographed tumors. Profiles of *pO*_2_, where the interstitial regime is delineated by the centroids of two adjacent blood vessels, show expected gradients whose slope is negative moving away from the first vessel, then eventually become positive moving toward the second vessel. The slope property in these experiments qualitatively matches the slope property in our data involving single vessels (negative slope moving away from the vessel). It is not clear to us, however, whether their *pO*_2_ profiles would provide a meaningful quantitative comparison with the analogous anti-pimonidazole intensity gradients we measure in our image data. More analysis is required to establish the compatibility of these two lines of evidence before *in vivo* studies like Helmlinger, *et al* could provide an empirical validation of our histological results, or vice-versa. Moreover, the authors make three findings of interest to our study: (1) they found no correlation between *pO*_2_ and blood flow rate; (2) they found no correlation between intravascular *pO*_2_ and blood flow rate; and (3) *pO*_2_ did not correlate with the two measured parameters used to compute blood flow rate—red blood cell velocity and vessel diameter (within respective specified ranges). Taken together, these findings highlight the admissibility of histology images as a source of data for measuring oxygen gradients. Although histology images represent single time points, and capture a range of vessel diameters conveying a range of possible blood flow rates, the evidence of oxygen gradients in these images is intact to the extent it can be measured in these images.

There has been recent progress in automated tumor segmentation on histological images, for example example by Wang, *et al* [22]. They developed a robust tumor segmentation technique and tested it on H&E and immunohistochemistry stain slides. Their method comprised a tissue architecture extraction approach and a tumor texture learning model. The tissue architecture extraction approach used a stain separation method and an unsupervised multistage entropy-based segmentation method, and the tumor texture learning uses a Markov random field image segmentation system. Their method allowed fine pixel based segmentation for small tissue samples. Their tissue domain was human lung tumors. For their purposes they defined three classes of tissue morphology: tumor, stroma, and a third catch-all category for lymphoid, inflammatory cells, and necrosis. Importantly, they did not try their method on anti-pimonidazole stain images, which, especially in low concentrations of anti-pimonidazole, render images that have strikingly low contrast. While their approach seems to us a promising texture-learning-based alternative to the simple intensity-based method we employ [23], it is unclear to us whether their method can perform effectively on anti-pimonidazole images and thus characterize chronic tumor hypoxia.

A number of recent computational studies [24–27] have employed statistical model checking algorithms to verify spatiotemporal logical propositions in biological systems. They used Probabilistic Bounded Linear Temporal Logic (PBLTL) to characterize phenomena of interest in: a fibroblast growth factor signaling model, circadian rhythm, yeast heterotrimeric G protein cycle control, and the HMGB1 signaling pathway in cancer. In one study, Grosu, *et al* [28] developed a system to tackle the problem of learning and detecting emergent behavior in networks of cardiac myocytes. They constructed a Linear Spatial-Superposition Logic (LSSL) formula that characterized spatial patterns such as spirals, whose multiscale spatial characterizations are learned through a classification process. Their system successfully detected the emergence of spiral patterns and hence the approaching state of fibrillation. In the spirit of these studies, we aim to develop a spatiotemporal logical proposition—composed of explicit image feature predicates—that captures at least some characteristics of chronic tumor hypoxia.

In addition, we construct a linear regression function that learns what hypoxia is in terms of estimated linear coefficients on the image feature terms. We adapt this method from our earlier work [29] in a different image processing domain where it showed promising results.

## Materials and Methods

### Experimental setup

Our study is based on experiments that demonstrate hypoxia arising in human colon cancer. In this experiment, 2 × 10^6^ human colon cancer cells were injected into both flanks of nude mice. When the tumor volume reached ∼1500 mm^3^ (∼4 weeks post-injection), pimonidazole was administered via intraperitoneal injection. Ninety minutes after pimonidazole administration mice were euthanized, the tumors were excised and immediately fixed in formalin. Slides were then prepared from sections 10 *μ*m apart, alternating between H&E and anti-pimonidazole stains. Mice were euthanized by carbon dioxide-induced narcosis. All animal work was approved by New York University Langone Medical Center Institutional Animal Care and Use Committee.

### H&E staining

**Fig 1:**
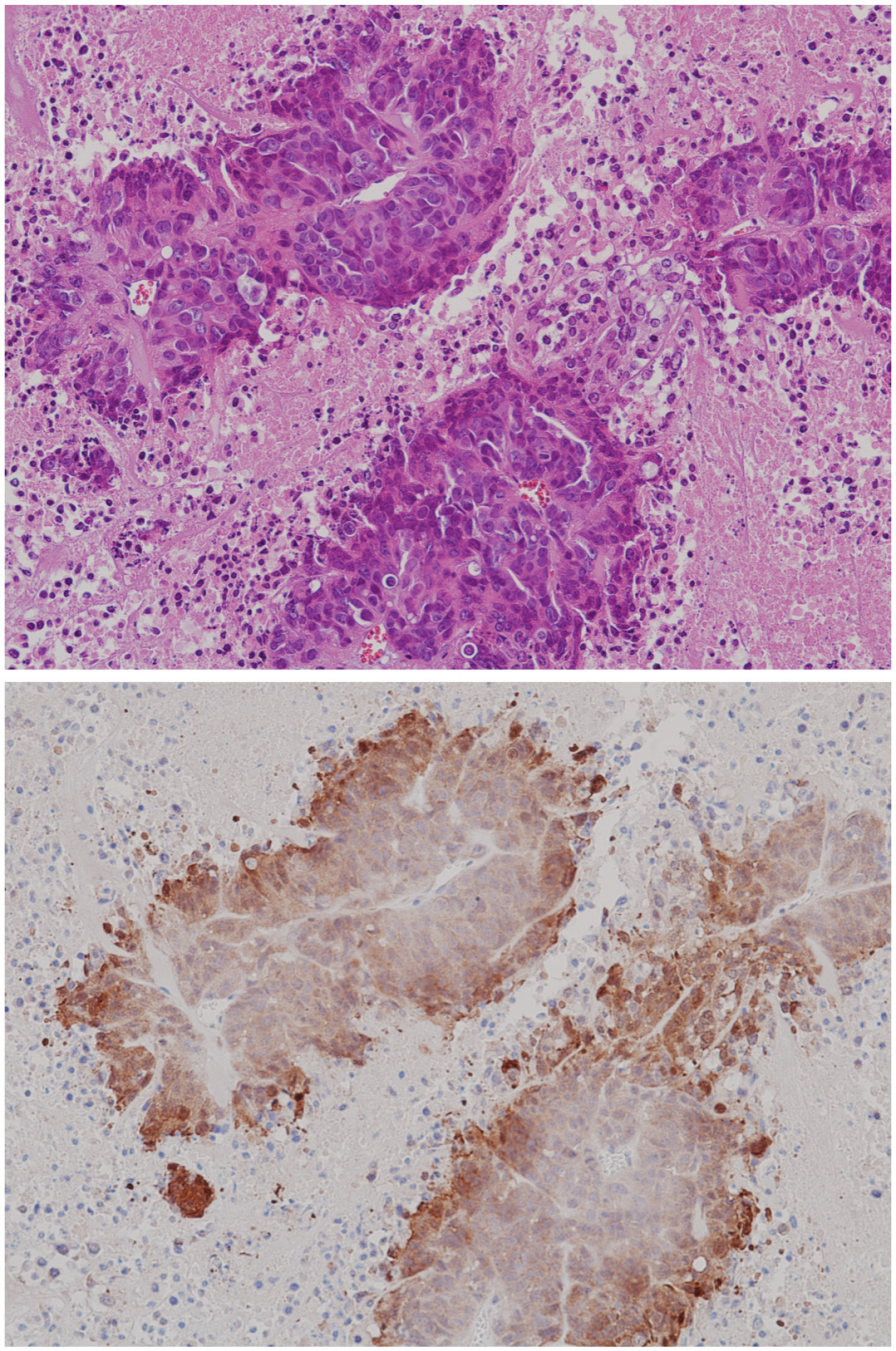
Histology stains. H&E (top) and anti-pimonidazole (bottom) stains of one of our study’s canonical tumor sections.

Hematoxylin and eosin stain (or “H&E stain”) is a common staining method in histology. It colors cell nuclei blue, then counterstaining colors non-nuclear, eosinophilic structures graded shades of orange, pink, and red. In our study, we use H&E stains of the tumor tissue for the primary purpose of locating blood vessels and for discriminating collagen. In Fig 1 (top), we see blood vessels appear within the boundary of the tissue as open lumens (white) populated with several to many red blood cells (small, bright pink spheroids). Collagen deposits appear as continuous structures (light pink) that infuse the tumor lesions and usually do not extend into the necrotic tissue (lightest pink, with interstitial spacing and much smaller, unenclosed nuclei).

### Anti-pimonidazole staining

Anti-pimonidazole staining is an immunohistochemical stain protocol used to detect and locate live cells undergoing hypoxia. In plasma, pimonidazole has a half-life of 25 minutes. It distributes to all tissues following injection, but it forms stable covalent adducts with thiol groups in proteins, peptides, and amino acids, only in those cells that have an oxygen concentration less than 14 micromolar (equivalent to a partial pressure pO_2_ = 10 mm Hg at 37 C). In the immunohistochemistry, anti-pimonidazole binds to these adducts allowing their detection. In addition to hypoxic regions in tumors, normal tissues of certain organs such as liver, kidney, and skin possess cells at or below pO_2_ of 10 mm Hg; these normal tissues, and only these, will bind pimonidazole. In Fig 1 (bottom) we see an anti-pimonidazole stain of one of our study’s canonical tumor sections. Hypoxic cells stain brown by degree of hypoxia. Notice the blood vessels are much more difficult to locate, though it is still possible. In most cases our procedure to locate vessels is to first manually register the H&E and anti-pimonidazole images (see note below), where the sections of the tumor are taken 10 *μ*m apart; second, locate the vessels on the H&E stain; then finally use this position on the anti-pimonidazole to approximate the vessel position, or to simply guide a more detailed examination of the anti-pimonidazole image until the vessel can be positively identified. Collagen complicates our study structurally and colorimetrically, which can be seen in the figure: collagen is difficult to distinguish from the necrotic tissue that surrounds the lesions.

### Aligning *Z*-stack images

To align two images, we match points in one image to corresponding points in the other to determine the displacement. In our H&E and anti-pimonidazole histology images we use blood vessel locations (the ones used above as centers of circular gradients) as our respective point sets, since these structures are easy to identify and match in both images. In S7 Fig we see the canonical anti-pimonidazole image with a vector field overlay. The three blue vectors denote the displacements of the three gradient centers. Each blue vector is labeled with *P*_*i*_ at the head (center position *i* in the anti-pimonidazole image) and *H*_*i*_ at the tail (the corresponding center position *i* in the H&E image). The vector lengths (in pixels) are labeled, as are the vector angles (in radians), measured relative to their respective dotted blue horizontal lines.

Notice the vector lengths and angles vary. If this were a straightforward image registration between identical, but translated, images, then we would expect the vectors to have identical lengths and angles. But several factors complicate the simple displacement alignment process. First, each image is a slice of an asymmetrical three-dimensional object undergoing morphological transformation. Second, the structures we chose are blood vessels, which presumably grow in directionally independent ways. Third, the microtome used to slice the tumor sample exerts nonuniform directional force upon the tissue. These and other, lesser, factors contribute to the complex transformation between the two images that involves translation, rotation, and scaling—yet even these taken together cannot account for the tissue’s morphological change between slices. If we assume rotation as a function of *x* and magnitude as a function of *y*, then we can fit two respective second-order polynomials to these three positional data with low error, and thereby create the vector field shown in red.

### Image analysis

Our approach consists in extracting qualitative and quantitative features from the histology images, namely the anti-pimonidazole stains. We classify these as: (1) features that derive from segmenting the image into the three tissue types depicted: viable tumor cells, hypoxic tumor cells, and necrotic tumor cells; (2) features related to the intra-lesion hypoxia gradient, as measured from radial distance away from the nearest vessel; (3) features that derive from multiscale analysis; and (4) features that relate to qualitative generalities about bounded and nested structure.

Once we have a set of image features, we proceed in two separate but related directions. First, we attempt to construct a logical proposition to describe hypoxia in space and time using an extension of Bounded Linear Temporal Logic (BLTL), whose primitives are image feature predicates. This is a human-driven process, following from human learning and generalization. Second, we attempt to construct a linear regression function that learns what hypoxia is in terms of estimated linear coefficients on the image feature terms. This is a machine-driven process, kept on the rails by a combination of false-positive and false-negative control, and feature dimensionality reduction where possible.

### Stratifying image data

For our initial examination of anti-pimonidazole images, the only selection criterion we applied was to keep to the interior of the tumor, away from its extremities. Since these are xenographed tumors, there are potentially many confounding factors at work near the interface between human tumor and mouse stroma. This was a baseline criterion, applied to all of the images we investigated, regardless of any further stratification. This gave us a set of 20 high-concentration anti-pimonidazole images, taken at 20× magnification, of various regions of the tumor interior. But as we became interested in the role vessels play in oxygenation of the tissue, we decided to further stratify the data, and select just those images whose 10× fields of view are ≥90% filled with non-necrotic cancer cells, and contain at least one blood vessel. This stratification gave us 8 such high-concentration anti-pimonidazole images, each taken at 10× and 20× magnification, having corresponding registered H&E images from a section 10 *μ*m away.

### Image preprocessing

We used Fig 1 as our canonical image for running examples. We did this for presentational convenience; our intuitions were developed examining many images, and our methods are applied to all specified images. The first step in our image preprocessing algorithm was to convert the RGB histology image into an 8-bit grayscale image. See S1 Fig (top).

Then we applied Gaussian smoothing (using a 5 × 5 mask and standard deviation of 5.0) iteratively until the high frequency structural information was averaged away (stopping at 100 iterations). See S1 Fig (bottom). We used no formal criteria for establishing these parameters, assuming that a consistent protocol for smoothing all images prior to downstream processing was more important than the degree of smoothness. We will address this issue in future work.

### Segmenting by histogram multithresholding

To get a qualitative feel for how we might identify tissue type by intensity level, we performed a preliminary investigation using two types of plot on our canonical image. When we viewed image intensity as a mesh plot (S2 Fig (top)), we observed three distinct planes of intensity in the image: necrotic tissue above, hypoxia tissue in the deepest recesses along the outer contour of the lesion, and viable (non-hypoxic) tissue rising up from that, but not to the height of the necrotic tissue. We also observed the backbone of collagen that runs along the middle of the lesion, and we were unable to distinguish collagen intensity levels from those of the necrotic tissue. We decided more information was given in the contour plot (S2 Fig (bottom)), where the proximity of equipotential curves conveys the steepness of the gradients in intensity.

To get a quantitative feel for how we might identify tissue type by intensity level, we examined image intensity histograms (S3 Fig). The histogram of the whole image showed a clear bimodal distribution, but selected sub-images showed a trimodal distribution. Using this distribution as a guideline, we segmented our canonical image into three non-overlapping intensity intervals: [0, 156] for hypoxic, [157, 175] for viable, and [176–255] for necrotic tissue, depicted as red-colored pixels in the top, middle, and bottom of S4 Fig, respectively. Naturally, because sharp thresholds truncate neighboring distributions, false-positive and false-negative cases are bound to emerge from this coarse approach. In the viable interval we saw false-positive outer contours around the hypoxic tissue, and the false-negative inner backbone areas where there are collagen deposits; and in the necrotic interval we saw false positive areas where collagen forms an inner backbone that partitions the viable tissue.

Since our canonical image is taken from a set of high-concentration anti-pimonidazole images, where the viable-hypoxic distinction is visually and numerically easier to make, we expected this intensity interval partition approach to perform worse on the low concentration anti-pimonidazole images, which we found (data not shown).

Given the cross-image variation we observed in the average intensity level for each tissue type, we became convinced that the manually-derived, fixed values we used for the intensity level partitions above could not be applied to all of our images. Thus we sought to use an adaptive approach, deciding on Otsu’s method [23] for automatic multiple thresholding, implemented in the Matlab Image Processing Toolkit as *multithresh*.

Despite the obvious Type I and Type II errors discussed above, we believed intensity-level-based segmentation could still be used to compare gross measures of viable-like and hypoxic-like cell areas within a whole image, and then provide characteristic ratios that could become image features.

### Measuring image intensity gradients

One of the most salient and consistent features of the anti-pimonidazole images under investigation is the presence of a gradient in the brown stain for hypoxia. In any given lesion, stain density is maximal at the outermost contour of the lesion, abutting necrotic tissue that surrounds it, and then diminishes steadily as a function of distance away from the extremity, toward the center (or central 1D spine) of the lesion. Equivalently, stain density decreases steadily as a function of radial distance away from the center (or orthogonally from the central 1D spine). The central area of a lesion is usually marked by a vessel.

#### The Intensity-Sample-Ray-Bundles algorithm

For our gradient measurement analysis, we designed an algorithm to perform radial intensity level sampling, along rays that extend from a given lesion center. One specifies three parameters: a center, (*x*_*c*_, *y*_*c*_), usually in the centroid of a blood vessel; *n*, the number of equal-angle-spaced rays that will sample the circle’s area; and *m*, the number of equal-angle-defined “bundles” (sectors) into which the rays will be considered for statistical analysis. For example, if *n* = 80, then a sample ray will be extended every $\frac{\pi }{40}$ radians, and if *m* = 1, then the rays that fall within 2*π* radians (all of the rays) will be considered for that bundle’s statistical analysis.

The image is first smoothed, as before. For a given ray, intensity level is sampled radially, from the inside out, until it encounters the edge of the image. One may specify (as optional parameters) the distance between samples along the ray, *d*_*s*_ in pixels (1 by default), and the square neighborhood radius, *r*_*n*_ in pixels, over which to average for that sample (0 by default since the image is already smoothed).

Once the samples have been taken along all of the rays, the rays are “stacked” and “sliced” in the following way. Each ray is an array or integers, whose index value (in the case of default value of *d*_*s*_) corresponds 1:1 to pixel distance away form the center. So if we “stack” all of the rays, aligning their array representations by their start index, we will have a measurement matrix, *M*, that has *m* rows and *c* columns, where $c=\frac{l_{max}}{d_{s}}$, and $l_{max}=\sqrt{x\_dim^{2}+y\_dim^{2}}$, the length of the hypotenuse of the triangle whose right angle sides are the *x* and *y* dimensions of the image being sampled. If *d*_*s*_ = 1 (by default), then *c* = *l*_*max*_. To see why *c* takes this value, consider the following extreme case we must be prepared to handle. If we place a center in one corner of the image, then a ray may extend to the opposite corner, requiring *l*_*max*_ array locations for its measurements.

Given *M*, we now compute mean, median, and standard deviation along column “slices” of *M*. This results in $\vec{mean}$, $\vec{median}$, and $\vec{std}$ vectors, whose array representation indices correspond to radial pixel distance away from the center. Since rays have different lengths—they each encounter the edge of the image in a different place, at a different distance from the center from the other rays—they each populate a row of *M* to a different extent, up to a certain column index; the remaining columns are populated with ∞ so that the part of our algorithm computing $\vec{mean}$, $\vec{median}$, and $\vec{std}$ knows when to drop this ray from the computation.

Now we compute the radius of the measurement area, *r*_*m*_, in the following way. One may specify (as an optional parameter) a threshold length, *l*_*t*_ (defaults to 1000 pixels), over which to locate the global minimum (darkest point) in $\vec{median}$. That is, $r_{m}=\min_{1\leq i\leq l_{t}}\left\{\vec{median}\left(i\right)\right\}$.

Our algorithm now creates three plots of the data, where the *x*-axis denotes distance from the center, and the *y*-axis denotes intensity level. The first shows every ray measurement (various colors), upon which $\vec{mean}$ (blue) and $\vec{median}$ (red) are overlaid; its title gives *r*_*m*_. The second shows $\vec{mean}$ (blue) ± $\vec{std}$ (gray), overlaid with segmented least squares fits to $\vec{mean}$ (black); its title gives the length (*l*), slope (*s*), and least squares error (*e*) for each fitted segment. The third shows $\vec{median}$ (red) ± $\vec{std}$ (gray), overlaid with segmented least squares fits to $\vec{median}$ (black); its title gives the length (*l*), slope (*s*), and least squares error (*e*) for each fitted segment. The segmented least square fits are given by a dynamic programming algorithm [30], using a cost parameter *C* = 200. We should note now that this entire process is bounded by, and repeated for, each bundle. So for example, if *m* = 4, then $\vec{mean}$, $\vec{median}$, $\vec{std}$, and *r*_*m*_ are computed, and plots are created, for those rays that fall within each successive $\frac{\pi }{2}$ of the circle.

**Fig 2:**
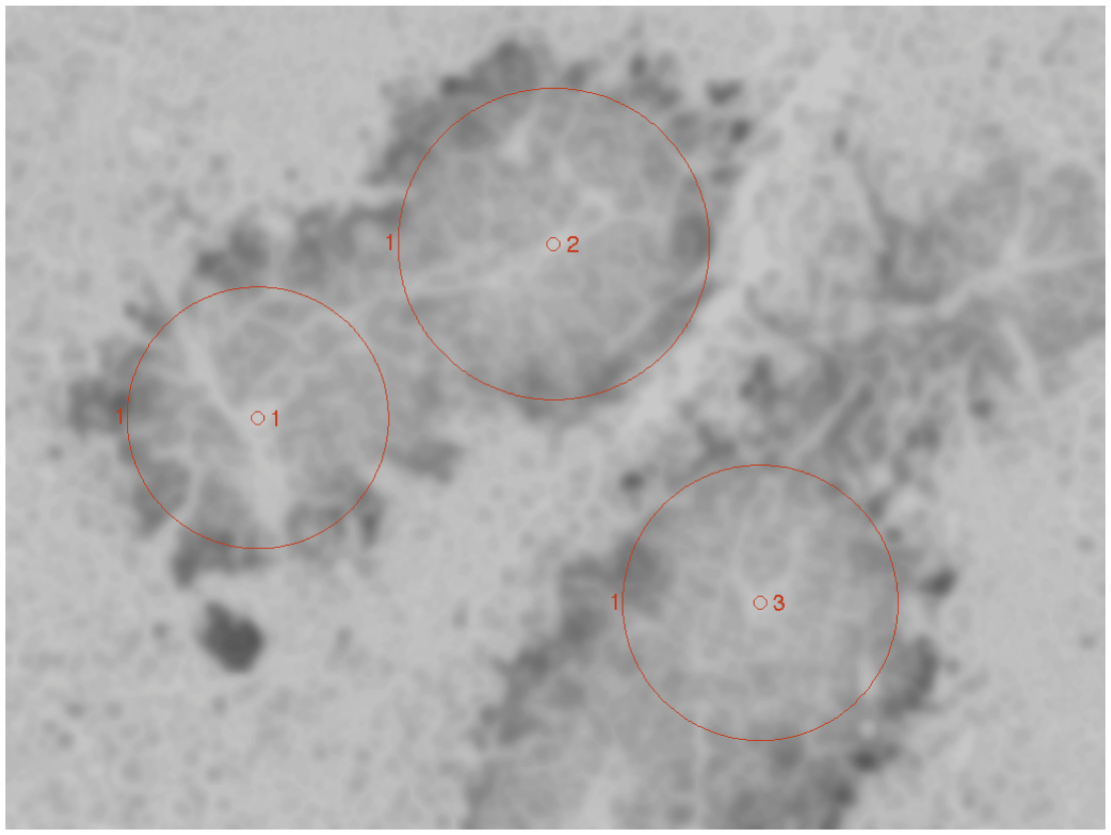
Loci of single-bundle hypoxia gradients. Circles (red) defined by the *r*_*m*_ found by the Intensity-Sample-Ray-Bundles algorithm for each of the three centers we specified, corresponding to vessel locations in the registered H&E image. Here we show *m* = 1 sector (2*π* radians per sector) for each center. Sectors are labeled with red numbers, counterclockwise, just outside of the red sector contour.

**Fig 3:**
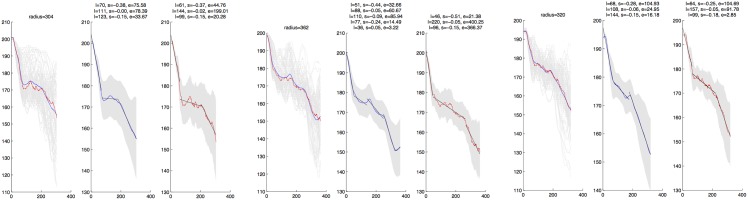
Hypoxia gradient analysis. Intensity level analysis produced by the Intensity-Sample-Ray-Bundles algorithm for centers 1 (left 3 panels), 2 (middle 3 panels), and 3 (right 3 panels). Intensity-Sample-Ray-Bundles creates three plots of the data, where the horizontal axis denotes distance from the center (pixels), and the vertical axis denotes intensity level. The first panel shows every ray measurement (light gray), upon which $\vec{mean}$ (blue) and $\vec{median}$ (red) are overlaid; its title gives *r*_*m*_ (pixels). The second panel shows $\vec{mean}$ (blue) ± $\vec{std}$ (gray), overlaid with segmented least squares fits to $\vec{mean}$ (black); its title gives the length (*l*, pixels), slope (*s*), and least squares error (*e*, pixels) for each fitted segment. The third panel shows $\vec{median}$ (red) ± $\vec{std}$ (gray), overlaid with segmented least squares fits to $\vec{median}$ (black); its title gives the length (*l*, pixels), slope (*s*), and least squares error (*e*, pixels) for each fitted segment. The segmented least square fits are given by a dynamic programming algorithm using a cost parameter *C* = 200.

Fig 2 shows the circles (red) defined by the *r*_*m*_ found for each of the three centers specified in our canonical image (*n* = 80, *m* = 1), corresponding to vessel locations in the registered H&E image. The intensity analysis for the three circles’ areas is given in Fig 3. S6 Fig shows the sectors (red) defined by the *r*_*m*_ found for each bundle of each of the three centers specified in our canonical image (*n* = 80, *m* = 8), corresponding to vessel locations in the registered H&E image. We do not show the corresponding 24 intensity analysis figures.

#### Stratifying image data with respect to gradients

Our first examination of high-concentration anti-pimonidazole images using this method was inconclusive. While it provided evidence for the presence of a gradient following the description above, the slopes of the relevant segments in the linear fit to the mean and median intensity measurements contained too much variation for a meaningful measurement of gradient steepness. It is common practice in many biology experiments to stain tissues using at least two concentration levels. The higher (or highest) concentration functions as a binary test for effectiveness of the stain. It answers: Is the phenomenon captured? Did it stain correctly? Provided that it did, follow up staining is conducted at lower concentrations. In the case of our data set, two concentrations, high and low, were used. Since the high-concentration images might contain excessive contrast, saturating the regions of hypoxia—beneficial for intensity-level-based image segmentation—this may swamp the more subtle gradient signal. We realized that we should attempt the same analysis on a corpus of low-concentration anti-pimonidazole images. For the purposes of measuring gradients, we sought to stratify the data differently than before, and select low-concentration anti-pimonidazole images, taken at 10× magnification, that contain one or more complete lesions, each containing one or more blood vessels. This gave us 23 such anti-pimonidazole images, each taken at 10× magnification, having corresponding registered H&E images from a section 10 *μ*m away.

### Measuring Quad-Tree statistics

To examine the property of intensity variance at different scales in the image, we employed the Quad-Tree algorithm, adapting it to work with any aspect ratio, not just square images. This works in the following way. For the given rectangle *R*, consider the set of pixels, *P*, within it, and the corresponding set of intensity values, *I*_*P*_. If the $CV\left(I_{P}\right)=\frac{\sigma (I_{P})}{\mu (I_{P})}>0.02$ then decompose *R* into four equal-size rectangles, *R*_1_, *R*_2_, *R*_3_, *R*_4_, and perform the quad-tree algorithm on *R*_1_, *R*_2_, *R*_3_, *R*_4_. This method quickly locates those regions of the image that contain a sufficiently high noise-to-signal ratio. S5 Fig shows the quad-tree decomposition of our canonical image.

We implemented a version of Quad-Tree, that we call Ply-Stats-Quad-Tree, that reports statistics related to the search tree for the image that it processes. These include the count, sum, mean, median, standard deviation, and coefficient of variation (CV) for the number of leaves at each ply, and a histogram of the counts of leaves at each ply. We use CV in intensity value of the current frame’s pixels as our splitting property, where CV exceeding a given threshold, *τ*, generates a split. The algorithm reports search tree statistics for the Quad-Tree dissection at a given value of *τ*.

### Deriving canonical EPC signatures

The Euler-Poincaré characteristic (EPC), one of the Minkowski functionals [31–33], is a measure of structural connectedness (or alternatively, porousness), and it has been used recently in two applications. The first concerns measuring bone density. Rath, *et al* [34] used the EPC to visualize and assess local trabecular bone structure; and Roque, *et al* [35] used the EPC to identify low bone density from vertebral tomographic images. The second application is in classifying tumors. Hutterer, *et al* [36] used the EPC to assign a characteristic signature curve to each AFM image of different tumor types, then used that curve as the basis of a classification method. We were intrigued by the use of characteristic EPC curves as an image feature by which to logically characterize, or functionally classify, chronic tumor hypoxia, and so apply this algorithm in our analysis.

We implemented an algorithm that follows directly from the approach taken by Hutterer, *et al* [36] to construct an Euler-Poincaré signature curve for an image. First, it converts the RGB image I to an 8-bit gray level image $I_{g}$, but does not smooth. Then for each gray level *i* = 1, …, 255, it produces a binary intensity-thresholded image $I_{i}$ and records $EPC(I_{i})$ for each *i*. This method gives a signature EPC curve for each I that could serve as an image feature.

### Spatiotemporal logical characterization of hypoxia in tumor histology

**Fig 4:**
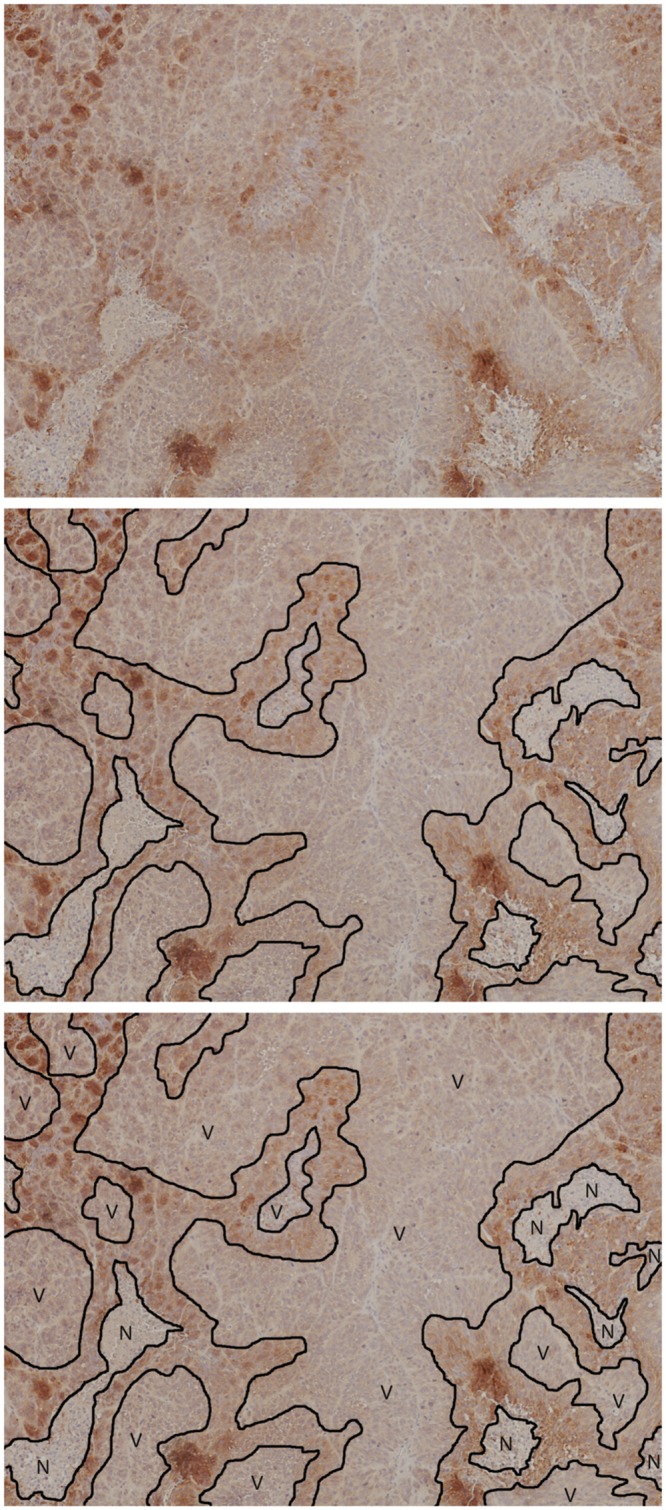
Tissue types by manual spatial partitioning. Another (unsmoothed) canonical anti-pimonidazole image (top), its manual partitioning (middle), and its labeled partitions (bottom). Key: V = viable, N = necrotic; unlabeled, brown regions are hypoxic.

Next we consider spatial partitioning, where continuous boundaries that separate tissue types are introduced into the image. This requires some degree of familiarity to manually parse these histology images, and so lacks the scalability in the number of images we require for statistical analysis. In Fig 4 we have another canonical anti-pimonidazole image, its manual partitioning, and its labeled partitions. Segmentation by partitioning reveals containment properties of the different regions and leads us to infer which tissue structures are nestable.

We first make some observations about the histological data that we can formulate as grammatical transformations. Then, in the results and discussion section, we use these transformations to globally constrain spatiotemporal logic predicates comprising the specific image processing features discussed above.

#### Using a grammar to describe how tissue regions transform

In Fig 4 (bottom), we segment and unambiguously identify viable (V), hypoxic (H), and necrotic (N) tissue regions in our anti-pimonidazole images. After identifying these regions on our full set of images, we observe the following qualitative patterns. Temporally: N always expands. Spatially: at any given time, in any given image, selecting a point and proceeding in a single direction away from the point will traverse either the V → H (ascending gradient) → N → H (descending gradient) → V cycle, or the V → H (ascending gradient → descending gradient) → V cycle; and the variation in the width of H, measured in the V → N direction, is much less than the variation in the width of the N or V regions measured in any direction—where N and V are blobs, H tends to be a well-defined band about V.

From these observations, we formulate the following two axioms for the tissue regions.

**A1 (spatial)** V and N are invalid neighbors; H must separate them.
**A2 (temporal)** There is a temporal monotonicity in how a region develops: V becomes H, and H becomes N, where N is the absorbing state.

From axioms *A1* and *A2*, we can derive both *context-free* and *context-sensitive* grammar production rules for the spatiotemporal transformation of hypoxia. The *context-free* production rules correspond to origination of a new tissue type, to nesting, to diversification. The *context-sensitive* production rules correspond to elimination of an existing tissue type, to collapsing, to homogenization. Axioms *A1* and *A2* lead us to derive *valid* production rules and restrict us from deriving *invalid* production rules.

Here are the four *valid* production rules:

1. V → V H V (H origination in V) by *A2*
2. H → H N H (N origination in H) by *A2*
3. H V H → H (V elimination in H) by *A2*
4. N H N → N (H elimination in N) by *A2*

Here are the eight *invalid* production rules:

1. H → H V H (V origination in H) by *A2*
2. N → N H N (H origination in N) by *A2*
3. N → N V N (V origination in N) by *A2*
4. V → V N V (N origination in V) by *A1*
5. H N H → H (N elimination in H) by *A2*
6. V H V → V (H elimination in V) by *A2*
7. N V N → N (V elimination in N) by *A1*
8. V N V → V (N elimination in V) by *A1*

#### Using a logic to describe hypoxia

We have defined above some quantitative and qualitative image features we now wish to incorporate into a logical proposition that describes what hypoxia is like in space and time. We will apply thresholds to the quantitative features to render them as predicates, and thus build up our final proposition out of these predicates.

#### Extending Probabilistic Bounded Linear Temporal Logic

The logic we develop here is an adaptation of Probabilistic Bounded Linear Temporal Logic (PBLTL) [24] that accommodates the three dimensions of space as well as time.

For a stochastic model simulation *S*, let the set of state variables *SV* be a finite set of real-valued variables. A Boolean predicate over *SV* is a constraint of the form *u* ∼ *v*, where *u* ∈ *SV*, ∼ ∈ { ≥, ≤, = }, and v∈R. A BLTL property is built on a finite set of Boolean predicates over *SV* using Boolean connectives and spatiotemporal operators. The syntax of the logic is given by the following grammar: $\phi ::=u\sim v|(\phi_{1}\vee \phi_{2})|(\phi_{1}\wedge \phi_{2})|\neg \phi_{1}|(\phi_{1}U^{\left\{x_{1},x_{2},x_{3},t\right\}}\phi_{2})$, where u∈SV,∼∈{≥,≤,=},v∈Q, and $x_{1},x_{2},x_{3},t\in Q_{\geq 0}$. We can define additional spatiotemporal operators such as $F^{\left\{x_{1},x_{2},x_{3},t\right\}}\psi =TrueU^{\left\{x_{1},x_{2},x_{3},t\right\}}\psi$ and $G^{\left\{x_{1},x_{2},x_{3},t\right\}}\psi =\neg F^{\left\{x_{1},x_{2},x_{3},t\right\}}\neg \psi$ in terms of the bounded until $U^{\left\{x_{1},x_{2},x_{3},t\right\}}$. A PBLTL formula is a one of the form *P*_≥*θ*_(*ϕ*), where *ϕ* is a BLTL formula and *θ* ∈ (0, 1). We say that *S* satisfies PBLTL property *P*_≥*θ*_(*ϕ*), denoted by *S* ⊨ *P*_≥*θ*_(*ϕ*), if and only if the probability that an execution of *S* satisfies BLTL property *ϕ* is greater than or equal to *θ*.

Let *x*_*d*_ denote the spatial dimension *x*_1_, *x*_2_, or *x*_3_ we wish to specify, and let *x*_*lim*_ and *t*_*lim*_ denote the limits in spatial dimension *x*_*d*_ and time dimension *t*, respectively, we wish to specify. The spatiotemporal operators can be interpreted as follows:

- $\phi_{1}U^{x_{d},x_{lim}}\phi_{2}$ means within *x*_*lim*_ spatial units in *x*_*d*_, *ϕ*_1_ holds until *ϕ*_2_ holds.
- $\phi_{1}U^{t,t_{lim}}\phi_{2}$ means within *t*_*lim*_ time units in *t*, *ϕ*_1_ holds until *ϕ*_2_ holds.
- $F^{x_{d},x_{lim}}\phi$ means within *x*_*lim*_ spatial units in *x*_*d*_, *ϕ* holds.
- $F^{t,t_{lim}}\phi$ means within *t*_*lim*_ time units in *t*, *ϕ* holds.
- $G^{x_{d},x_{lim}}\phi$ means for *x*_*lim*_ spatial units in *x*_*d*_, *ϕ* holds.
- $G^{t,t_{lim}}\phi$ means for *t*_*lim*_ time units in *t*, *ϕ* holds.

Continuing to follow Jha, *et al* [24], we define the semantics of our extended BLTL with respect to executions of *S*. Let *σ* ⊨ *ϕ* denote that an execution trace *σ* of *S* satisfies *ϕ*. Let *σ* = (*s*_0_, *t*_0_), (*s*_1_, *t*_1_), … be an execution of the simulator along states *s*_0_, *s*_1_, … with durations $t_{0},t_{1},...\in R$. We denote the execution trace starting with state *i* by *σ*^*i*^. The value of the state variable *x* in *σ* at state *i* is denoted by *V*(*σ*, *i*, *x*). The semantics of our extended BLTL for a trace *σ*^*k*^ starting at the *k*^*th*^ state (k∈N) is defined as follows:

- *σ*^*k*^ ⊨ *x* ∼ *v* iff *V*(*σ*, *k*, *x*)∼*v*
- *σ*^*k*^ ⊨ *ϕ*_1_ ∨ *ϕ*_2_ iff *σ*^*k*^ ⊨ *ϕ*_1_ or *σ*^*k*^ ⊨ *ϕ*_2_
- *σ*^*k*^ ⊨ *ϕ*_1_ ∧ *ϕ*_2_ iff *σ*^*k*^ ⊨ *ϕ*_1_ and *σ*^*k*^ ⊨ *ϕ*_2_
- *σ*^*k*^ ⊨ ¬*ϕ* iff *σ*^*k*^ ⊨ *ϕ* does not hold
- $\sigma^{k}⊧\phi_{1}U^{x_{d},x_{lim}}\phi_{2}$ iff ∃i∈N such that (1) ∑_0<*l* ≤ *i*_(*x*_*d*, *k*+*l*_ − *x*_1, *k*+*l*−1_) ≤ *x*_*lim*_, (2) *σ*^*k*+*i*^ ⊨ *ϕ*_2_, and (3) for each 0 ≤ *j*<*i*, *σ*^*k*+*j*^ ⊨ *ϕ*_1_.
- $\sigma^{k}⊧\phi_{1}U^{t,t_{lim}}\phi_{2}$ iff ∃i∈N such that (1) ∑_0 ≤ *l* < *i*_ *t*_*k*+*l*_ ≤ *t*_*lim*_, (2) *σ*^*k*+*i*^ ⊨ *ϕ*_2_, and (3) for each 0 ≤ *j* < *i*, *σ*^*k*+*j*^ ⊨ *ϕ*_1_.

Each of the last two semantic statements has three necessary conditions, which we clarify as follows. (1) In the case of spatial units, the sum of the spatial intervals in *x*_*d*_ along the state sequence *k*, *k*+1, …, *k*+*i* should be less than or equal to the limit value of *x*_*lim*_ specified—this implements “within *x*_*lim*_ spatial units in *x*_*d*_.” In the case of time units, the sum of durations along the state sequence *k*, *k*+1, …, *k*+*i* should be less than or equal to the limit value of *t*_*lim*_ specified—this implements “within *t*_*lim*_ time units.” (2) This implements “at some state *i* beyond state *k*, *ϕ*_2_ holds.” (3) This implements “For each state from *k* up to but not including state *i*, *ϕ*_1_ holds.”

### Linear regression functional characterization of hypoxia in tumor histology

We now propose a second way to characterize hypoxia in tumor histology, using a simple machine learning approach to adaptively weigh the contributions of each and every image processing feature to score candidate histology images (or simulation results) for their similarity to ones containing stable local regions of hypoxia.

#### Ergodic assumption

We assume the chronic tumor hypoxia process that generates our image data to be ergodic: since we see so many instances of lesions, we are likely seeing every temporal state of a typical lesion, and so, in the limit of static images, we observe the *temporal* and *spatial* phenomenon of hypoxia.

#### Linear regression learning

We would now like to incorporate the quantitative image features defined above into a linear functional form, whose weights are learned by regression [37], for a lesion hypoxia similarity metric. Our approach here is adapted from earlier work in a different domain [29]. This entails solving an overdetermined system of equations, given by *a*_1_ *f*_1, *j*_+…+*a*_*n*_ *f*_*n*, *j*_ = 1, where the *a*_*i*_, *i* = 1, …, *n* are the *n* feature coefficients to be learned and the *f*_*i*, *j*_, *i* = 1, …, *n*, *j* = 1, …, *m* are the corresponding *n* feature values over *m* ≥ *n* observations forming the feature matrix, *F*. We train a linear regression model on *m* calibrating lesions, having known similarity score 1, using values from the *n* features, giving $F\vec{a}=\vec{1}$. The model has the analytic solution $\vec{a}={\left(F^{T}F\right)}^{-1}F^{T}\vec{1}$. This gives a trained similarity estimator, $S_{T}=a_{1}f_{1}+...+a_{n}f_{n}$.

This formulation of $S_{T}$ assumes all lesions, i.e. their associated feature values, have equal weight, owing to their equivalent validity as observations. However, such an assumption may be challenged on the grounds that upon taking into consideration the difference between the empirically measured null distribution and the actual shape of the distribution in feature measurements, certain observations appear to be false positives, and others false negatives—a notion formally addressed by robust regression, namely, the Beaton-Tukey formulation.

#### Weighting training data to address Type I and Type II errors

Normally, false positive examples appear as ones that deviate significantly from the null-distribution, and if not discarded, can affect the statistical estimators adversely. However, instead of discarding such outliers using sharp-thresholds, and using the filtered examples in the estimator, one may assign to each data point a positive weight that signifies how likely it is that a particular example is an outlier. Such a weighting scheme could be based on the ideas underlying robust M-estimators—a class of central tendency measures that make them resistant to local misbehavior caused by outliers (e.g., false positives). We adapted the Beaton-Tukey biweight [38]—an iteratively reweighted measure—for this purpose of central tendency. We note that other schemes, such as Huber’s M-estimator, could have been used with similar performance. Both the biweight and the Huber weight functions are available in standard statistical packages. Here we use Matlab’s *robustfit* command with default parameters (weight function “bisquare,” using a tuning constant of 4.685).

In the context of our system, the *x*_*i*_, *i* = 1, …, *m* are the feature values of the *m* calibrating lesions in the training set. Each lesion is assigned a weight, *w*_*i*_. If its weight is zero, then the corresponding lesion is discarded from the training set. Of the *m* training molecules, *m*′ remain. This gives a weighted-trained similarity estimator, $S_{W}=a_{1}f_{1}+...+a_{n}f_{n}$.

In our modeling of estimation error above, one or more features in training may introduce too much variance (systematic error) or dependence (model error). We would like our model to have an extensible and adaptive structure, where any number of features may be used, and proceed with confidence, knowing that noisy or dependent features will have a contribution to the estimate that shrinks to zero. We now apply one of the following patterns of shrinkage to the feature coefficients, $\vec{a}$.

#### Shrinking feature coefficients to reduce feature space dimensionality

In 1961, James and Stein published their seminal paper [39] describing a method to improve estimating a multivariate normal mean, $\vec{\mu }=\left[\mu_{1},...,\mu_{k}\right]$, under expected sum of squares error loss, provided the degree of freedom *k* ≥ 3, and the *μ*_*i*_ are close to the point to which the improved estimator shrinks.

When extreme *μ*_*i*_ are likely, then spherical shrinkage may give little improvement. This may occur, for instance, when the *μ*_*i*_ arise from a prior distribution with a long tail. A property of spherical shrinkage is that its performance is guaranteed only in a small subspace of parameter space, requiring that one select an estimator designed with some notion of where $\vec{\mu }$ is likely to be, such that the estimator shrinks toward it. An extreme *μ*_*i*_ will likely be outside of any small selected subspace, implying a large denominator and so little, if any, shrinkage in $\vec{a}$, thereby giving no improvement. To address this problem, Stein proposed a coordinate-based (or truncated) shrinkage method.

#### Applying the metric

Once the weighted-trained model feature coefficients, *a*_*i*_, have undergone shrinkage, $a_{i}^{\prime }$, we have our final hypoxia similarity estimator, $S_{W}=a_{1}^{\prime }f_{1}+...+a_{n}^{\prime }f_{n}$ that can measure out-of-sample lesions for their similarity to hypoxic lesions. $S_{W}$ gives a [0, 1] numerical score instead of a {0, 1} outcome. A simulator that implements this scoring function can then feed a branch-and-bound (optimization) process that can explore the simulator’s configuration parameter space.

### Code availability

All code described in this paper is written in Matlab and available in the GitHub repository: https://github.com/aesundstrom/tumor-hypoxia-image-processing

### Histology image availability

All histology images of chronic tumor hypoxia used in this paper are available in the Harvard Dataverse: http://dx.doi.org/10.7910/DVN/SI32FV

## Results and Discussion

First, we discuss four experiments corresponding to the four image processing features we develop here—segmenting by histogram multithresholding, measuring image intensity gradients, measuring quad-tree statistics, and deriving canonical EPC signatures. Next, we use these image processing features to develop a spatiotemporal logical characterization of chronic tumor hypoxia in histology images. Finally, we propose another way to characterize chronic tumor hypoxia in histology images, using a simple machine leaning approach.

### Segmenting by histogram multi-thresholding

#### Setup

**Fig 5:**
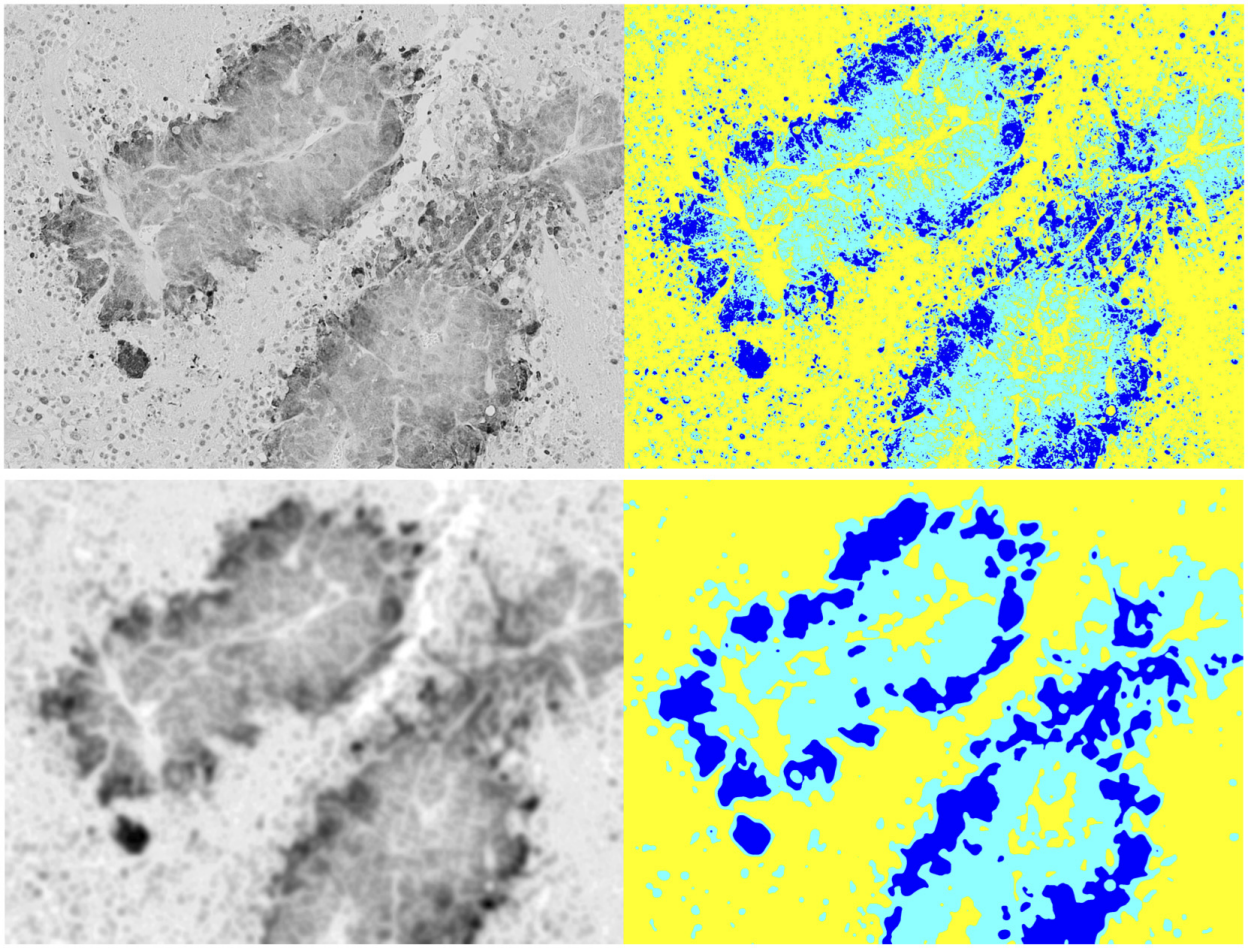
Otsu segmentation and smoothing. How Otsu’s multithreshold segmentation differs between unsmoothed gray (upper left) and smoothed gray (lower left) images. Corresponding images on the right show dark blue regions that denote hypoxic cells, light blue regions that denote viable cells, and yellow regions that denote necrotic cells.

We applied Otsu’s method to multithreshold a set of *n*_*T*_ = 66 images across stratification criteria, magnification, and high and low concentrations of anti-pimonidazole. To distinguish between results for the high- and low-concentration images, we place, alongside the results for the total set of images, those results for *n*_*H*_ = 36 high-concentration images and *n*_*L*_ = 30 low-concentration images, computed separately. See Table 1. The table organization also reflects the distinction between unsmoothed and smoothed gray images. We illustrate this distinction in Fig 5.

**Table 1:** Otsu’s multithreshold segmentation of unsmoothed versus smoothed images over the *total* set of images (*n*_*T*_ = 66), *high* anti-pimonidazole images (*n*_*H*_ = 36), and *low* anti-pimonidazole images (*n*_*L*_ = 30). We report pixel areas as proportions of the entire set of pixels in the image (*I*); hence *H*:*I*, *V*:*I*, and *N*:*I*. We also report another proportion of interest, namely that of hypxic to viable cells in the image, *H*:*V*.

*n*_*T*_ = 66, *n*_*H*_ = 36, *n*_*L*_ = 30	*μ*_*T*_	*σ*_*T*_	*CV*_*T*_	*μ*_*H*_	*σ*_*H*_	*CV*_*H*_	*μ*_*L*_	*σ*_*L*_	*CV*_*L*_
unsmoothed partition 1	136.23	12.25	0.09	143.47	10.74	0.07	127.53	7.31	0.06
unsmoothed partition 2	158.06	13.58	0.09	166.89	11.44	0.07	147.47	6.56	0.04
unsmoothed *H*:*I* pixels	0.14	0.04	0.27	0.14	0.03	0.25	0.15	0.04	0.29
unsmoothed *V*:*I* pixels	0.40	0.07	0.17	0.42	0.05	0.12	0.38	0.08	0.20
unsmoothed *N*:*I* pixels	0.46	0.09	0.20	0.45	0.07	0.16	0.47	0.11	0.23
unsmoothed *H*:*V* pixels	0.36	0.08	0.24	0.32	0.07	0.20	0.39	0.09	0.22
smoothed partition 1	144.76	13.13	0.09	150.39	14.11	0.09	138.00	7.55	0.05
smoothed partition 2	158.03	13.91	0.09	166.19	13.03	0.08	148.23	6.80	0.05
smoothed *H*:*I* pixels	0.21	0.09	0.41	0.16	0.06	0.38	0.27	0.07	0.27
smoothed *V*:*I* pixels	0.41	0.07	0.16	0.41	0.08	0.19	0.41	0.05	0.13
smoothed *N*:*I* pixels	0.38	0.11	0.29	0.43	0.11	0.26	0.33	0.08	0.25
smoothed *H*:*V* pixels	0.52	0.23	0.45	0.39	0.14	0.37	0.67	0.22	0.33

#### Results

In Table 1 we observed the following for unsmoothed and smoothed images. Otsu’s method found intensity level partitions whose means are remarkably stable (*CV* = {0.09, 0.09}, {0.09, 0.09}) across such a variable total set of images. As expected, the stability of these partitions increased as we stratified the images into high-concentration (*CV* = {0.07, 0.07}, {0.09, 0.08}) and low-concentration (*CV* = {0.06, 0.04}, {0.05, 0.05}) subsets. Of the pixel proportions, the most stable mean value was always *V*:*I*, for the total set and both strata. The mean *H*:*V* ratio was also stable across strata (*CV* = {0.24, 0.20, 0.22}) for unsmoothed images, but not as much (*CV* = {0.45, 0.37, 0.33}) for smoothed images. In unsmoothed images, across strata the *H*:*V* ratio had a similar mean value (*σ* = {0.36, 0.32, 0.39}); in unsmoothed images, across strata, the mean values varied significantly (*σ* = {0.52, 0.39, 0.67}); between unsmoothed and smoothed images the corresponding mean *H*:*V* ratio values seemed to have no relationship ({0.36, 0.52}, {0.32, 0.39}, {0.39, 0.67}), though the smoothed, high-concentration mean value (0.39) did seem to fit with the cross-strata values in the unsmoothed images.

#### Discussion

The mean partition values, and the mean *H*:*V* ratio values for unsmoothed images, were stable. They could serve as image features.

### Measuring image intensity gradients

#### Setup

We used our Intensity-Sample-Ray-Bundles algorithm to measure gradient properties on high- and low-concentration anti-pimonidazole images that adhere to the stratification criterion that they contain at least one complete lesion at 10× magnification, and the lesions contain at least one blood vessel. For each image, we specified one or more landmarks, (*x*, *y*) coordinates, that coincide with vessel locations on the corresponding H&E tumor sections (separated orthotopically by 10 *μ*m). These landmarks were passed to the algorithm to be used as centers from which to extend intensity sample rays. We measured all gradients using 80 intensity sample rays per circle, centered at each landmark. We selected 9 high-concentration anti-pimonidazole images (containing 25 landmarks) and 8 low-concentration anti-pimonidazole images (containing 29 landmarks).

For each landmark the algorithm explored, it outputted the mean and median intensity levels as a function of the radial distance away from the landmark. Both curves were optimally fit using segmented least squares, given by a dynamic programming algorithm [30], with a cost parameter *C* = 200. These curves were each usually fit by one, two, or three segments, of different lengths and slopes. These were superimposed on their respective mean and median curves as part of the output. (See Fig 3, for example.) In each case, we examined the output and selected either the mean or median curve fit, depending on which fit gave fewer segments; if they gave the same number of segments, then we selected the mean curve fit.

Since the length and slope of these fits characterizes the measured gradient, we would like to use these—actually the average of these, over as wide a sample as possible—as image features. However, we cannot compare, say, a one-segment fitted curve to a three-segment fitted one, since these give distinct characterizations of the gradient and we ought to respect that observed distinction. Because of this, we report our results in six tables. The one-, two-, and three-segment fits for the high-concentration anti-pimonidazole images, and the one-, two-, and three-segment fits for the low-concentration anti-pimonidazole images. See Tables 2, 3, 4, 5, 6 and 7.

**Table 2:** 1-segment radii in *high* anti-pimonidazole images. The values of *n*_*g*_ and *n*_*i*_ report that the statistics are from a sample of 2 gradients found in 1 image.

*n*_*i*_ = 1, *n*_*g*_ = 2	*μ*	*σ*	*CV*
radius	618.50	70.00	0.11
segment 1 length	459.00	11.31	0.02
segment 1 slope	-0.02	0.00	0.00
segment 1 error	348.38	70.70	0.20

**Table 3:** 2-segment radii in *high* anti-pimonidazole images. The values of *n*_*g*_ and *n*_*i*_ report that the statistics are from a sample of 7 gradients found in 4 images.

*n*_*i*_ = 4, *n*_*g*_ = 7	*μ*	*σ*	*CV*
radius	457.86	113.23	0.25
segment 1 length	84.86	35.41	0.42
segment 1 slope	-0.13	0.08	0.63
segment 1 error	106.57	126.50	1.19
segment 2 length	351.71	72.17	0.21
segment 2 slope	-0.03	0.02	0.50
segment 2 error	322.89	169.55	0.53

**Table 4:** 3-segment radii in *high* anti-pimonidazole images. The values of *n*_*g*_ and *n*_*i*_ report that the statistics are from a sample of 16 gradients found in 8 images.

*n*_*i*_ = 8, *n*_*g*_ = 16	*μ*	*σ*	*CV*
radius	477.25	95.07	0.20
segment 1 length	80.75	38.44	0.48
segment 1 slope	-0.22	0.12	0.56
segment 1 error	68.39	57.47	0.84
segment 2 length	219.00	85.48	0.39
segment 2 slope	-0.04	0.08	1.85
segment 2 error	158.19	123.14	0.78
segment 3 length	164.38	73.49	0.45
segment 3 slope	-0.09	0.06	0.63
segment 3 error	115.89	122.65	1.06

**Table 5:** 1-segment radii in *low* anti-pimonidazole images. The values of *n*_*g*_ and *n*_*i*_ report that the statistics are from a sample of 4 gradients found in 2 images.

*n*_*i*_ = 2, *n*_*g*_ = 4	*μ*	*σ*	*CV*
radius	348.00	49.29	0.14
segment 1 length	348.00	49.29	0.14
segment 1 slope	-0.09	0.03	0.30
segment 1 error	288.37	199.55	0.69

**Table 6:** 2-segment radii in *low* anti-pimonidazole images. The values of *n*_*g*_ and *n*_*i*_ report that the statistics are from a sample of 20 gradients found in 8 images.

*n*_*i*_ = 8, *n*_*g*_ = 20	*μ*	*σ*	*CV*
radius	454.55	138.59	0.30
segment 1 length	172.25	82.96	0.48
segment 1 slope	-0.21	0.19	0.91
segment 1 error	94.34	82.17	0.87
segment 2 length	267.40	125.64	0.47
segment 2 slope	-0.06	0.03	0.55
segment 2 error	112.97	111.27	0.98

**Table 7:** 3-segment radii in *low* anti-pimonidazole images. The values of *n*_*g*_ and *n*_*i*_ report that the statistics are from a sample of 5 gradients found in 4 images.

*n*_*i*_ = 4, *n*_*g*_ = 5	*μ*	*σ*	*CV*
radius	677.80	390.88	0.58
segment 1 length	153.60	74.42	0.48
segment 1 slope	-0.17	0.21	1.23
segment 1 error	61.13	37.22	0.61
segment 2 length	187.40	64.40	0.34
segment 2 slope	-0.08	0.07	0.94
segment 2 error	64.03	63.07	0.98
segment 3 length	318.20	417.14	1.31
segment 3 slope	-0.04	0.01	0.35
segment 3 error	141.12	276.56	1.96

We should note two considerations we made for selecting results to show here. First, we sometimes omitted spurious short or positive-slope segments that appeared first in the sequence of segments (i.e., closest to the center of the circle), since these constitute noisy measurements, usually due to the landmark residing in the center of a high-intensity lumen or some low-intensity blob of pixels; consequently, in some of the tables, the mean segment lengths do not sum to the mean radius length, owing to the mean length of the omitted segments. Second, we selected only gradients that corresponded to radii discovered by our algorithm whose length scales matched those of the lesions in which they resided, i.e., the contour of the circle defined by the radius coincides with the outermost contour of the hypoxic region in the lesion. (See Fig 2, for example.)

#### Results

For ease of discussion, let *H* denote the set of high-concentration anti-pimonidazole images or the segmented gradient curves that derive from them, and *L* denote the set of low-concentration anti-pimonidazole images or the segmented gradient curves that derive from them. Tables 2, 3 and 4 show the results for *H* images that have 1-, 2-, and 3-segment fits, respectively; and Tables 5, 6 and 7 show the results for *L* images that have 1-, 2-, and 3-segment fits respectively.

We immediately observed that the majority of gradients in *H* images had 3-segment fits, whereas the majority of gradients in *L* images had 3-segment fits. This less complicated structure of *L* gradients agreed with our intuition that they are better for characterizing continuous gradients that are not punctuated by the relatively flat middle segments we see with the *H* gradients. This was further bolstered by a closer examination of the structure of the segmented curves. In comparing the *H* vs *L* segmented gradient curves: *H* 1-segment curves were flatter than those of *L*; *H* 2-segment curves were flatter in both segments than those of *L*; and *H* 3-segment curves were defined by a concave-then-convex shape, whereas those of *L* were decidedly concave, i.e., they tended to have monotonically decreasing slopes as a function of radial distance away from the vessel.

Suppose we focus only on *L* gradient curves, believing they more closely reflect real underlying hypoxia gradients. We observed that as radial distance grows, the gradient became nonlinear, following from its concavity. We have not performed nonlinear fits to the gradient curves but suspect a quadratic (and certainly an exponential) curve would easily fit with low error.

As a proportion of their sum, the first and second *L* segments tended to be 0.39 and 0.61 of their total 2-segment length, respectively; and the first, second, and third *L* segments tended to be 0.23, 0.28, and 0.48 of their total 3-segment length, respectively.

#### Discussion

These segment proportions, their slopes, and the parameterized nonlinear fit to the gradient curve could serve as image features.

That said, we should note the statistical significance, and the degrees of error, we observed in the *L* gradient measurements. First, with 1-, 2-, and 3-segment sample sizes of 4, 20, and 5, respectively, we acknowledge that at least the 1- and 3-segment data were less than statistically significant, and even the extreme variance—particularly with the slopes of the first and second segments (*CV* = 1.23 and *CV* = 0.94, respectively), and the length of the third segment (*CV* = 1.31) in the 3-segment fit—these might have diminished with a larger sample. Looking at the more significant sample of 2-segment gradient measurements, we also observed high variance in the slopes, with *CV* = 0.91 and *CV* = 0.98 for segments 1 and 2, respectively. With this in mind, we are unclear how ultimately useful these measure would be as generalized, canonical features. Perhaps the parameterized nonlinear fit would be a more stable and therefore a more suitable feature.

**Fig 6:**
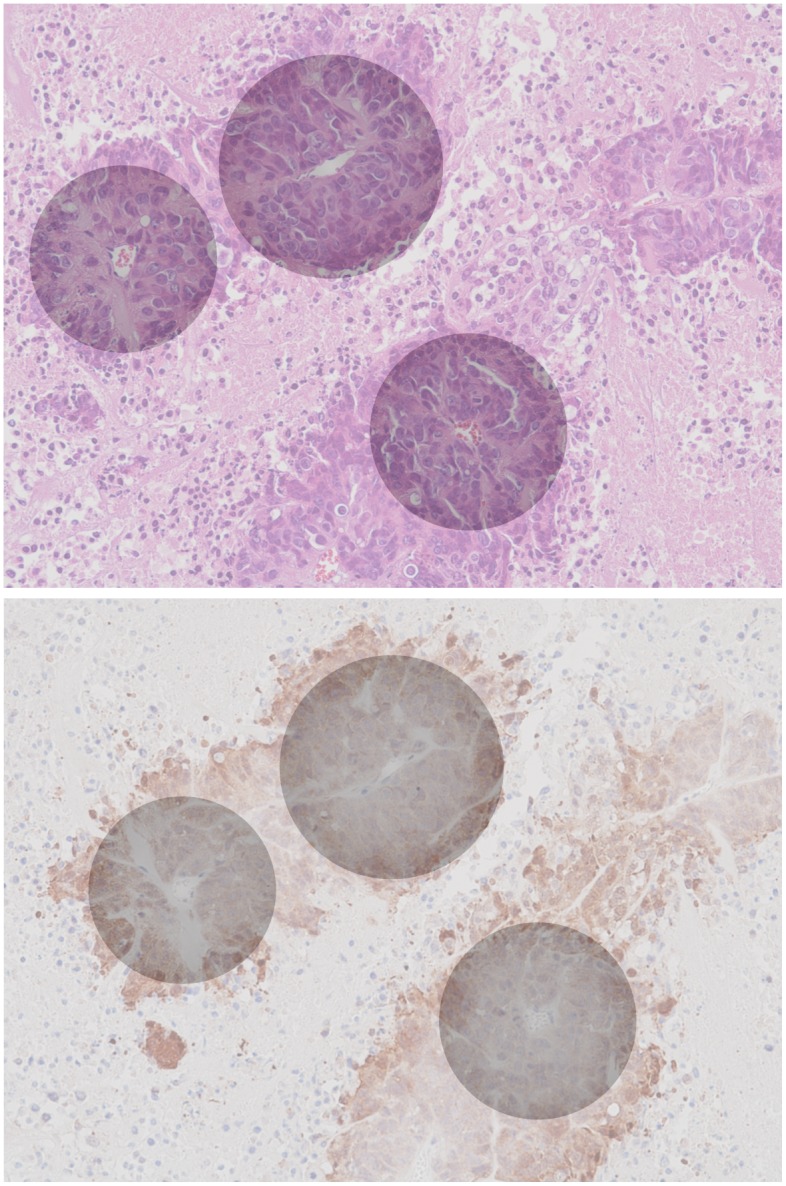
Synthetic histology. Inferred hypoxia gradients (gray) superimposed onto the canonical raw H&E (top) and anti-pimonidazole (bottom) images at half-opacity. Note that the positions of the gradient centers have been corrected as per our earlier observation regarding adjacent image registration (see S7 Fig).

Even with these limitations, one can create synthetic images by superimposing measured gradients on the original raw images. We illustrate this as follows. Using the segmented least-squares fits to the gradient functions measured in S1 Fig (bottom) and presented in Fig 3, we superimpose the corresponding gradients upon the raw anti-pimonidazole and H&E images (see Fig 6). Here, concentrically-plotted gray levels mirror the respective measured gradient values. The latter half-opacity (*α* = 0.5) synthesized image nondestructively combines the inferred information from the anti-pimonidazole image and the high-contrast structural information from the H&E image into a single view. These synthetic images could serve as a diagnostic tool in a clinical setting.

### Measuring quad-tree statistics

#### Setup

We used our Ply-Stats-Quad-Tree algorithm to compute statistics for *τ* ∈ [0.01, 0.10] by increments of 0.01, therefore generating 10 sets of statistics for a given image. We selected *τ* ∈ {0.1, 0.5, 0.9} to report here.

#### Results

We computed this for a set of *n* = 66 images across stratification criteria, magnification, and high and low concentrations of anti-pimonidazole. Anticipating a likely distinction between results for the high- and low-concentration images, we produce, along with the figure reporting the results for the total set of images (S8 Fig, left 3 panels), those results for *n* = 36 high-concentration images (S8 Fig, middle three panels), and *n* = 30 low-concentration images (S8 Fig, right three panels), computed separately. In each set of three panels, the first, second, and third panels show mean histograms for *τ* ∈ {0.1, 0.5, 0.9}, respectively.

#### Discussion

There is an overwhelming degree of error in these measures, regardless of stratification. We had hoped the mean histogram of ply counts (image frame sizes) might serve as an image feature, but while decreasing *τ* consistently produces a more stable mean profile, even the minimum value of *τ* = 0.01 we tested is too disperse, so frame size profiles are unusable as an image feature.

### Deriving canonical EPC signatures

#### Setup

We produced EPC signature curves for each image in a set of *n*_*T*_ = 66 images across stratification criteria, magnification, and high and low concentrations of anti-pimonidazole. We distinguished between results for the high-concentration (*n*_*H*_ = 36) and low-concentration (*n*_*L*_ = 30) images, stratified the resulting curves, and for each stratum, we computed a mean EPC curve separately. We plotted these canonical stratum curves with their respective standard deviations in S9 Fig (top).

Then we approximated each canonical stratum curve with an optimized segmented least-squares fit, and thereby compressed the data into a smaller number of real-valued coefficients—slope and *y*-intercept for each segment—that could potentially serve as image features. The segmented least square fits were given by a dynamic programming algorithm [30], using a cost parameter *C* = 50000. We also reported the sum of least-squares errors over all of the segments in the fit in S9 Fig (bottom). The segment fit coefficients and the error could potentially serve as image features.

#### Results

In S9 Fig (top) we show the mean EPC curve over the *total* set of images (left, *n* = 66), the *high* concentration anti-pimonidazole images (middle, *n* = 36), and the *low* concentration anti-pimonidazole images (right, *n* = 30).

We show the segmented least-squares fits to the mean EPC curves are given in S9 Fig (bottom). The fit on the left is composed of 18 segments (specified by 36 coefficients), giving a compression factor of 7.08 and a normalized least-squares error of 1082.84. The fit in the middle is composed of 18 segments (specified by 36 coefficients), giving a compression factor of 7.08 and a normalized least-squares error of 933.19. The fit on the right is composed of 21 segments (specified by 42 coefficients), giving a compression factor of 6.07 and a normalized least-squares error of 1063.19.

#### Discussion

Here too there is an overwhelming degree of error in these measures, regardless of stratification. While one can roughly discern a characteristic shape similarity in the curves, this is not a rigorously established feature, so EPC curves are unusable.

### Spatiotemporal logical characterization of hypoxia in tumor histology

Using our extended BLTL, we derive a preliminary spatiotemporal proposition of hypoxia in terms of the hypoxia image features discussed here.

**Fig 7:**
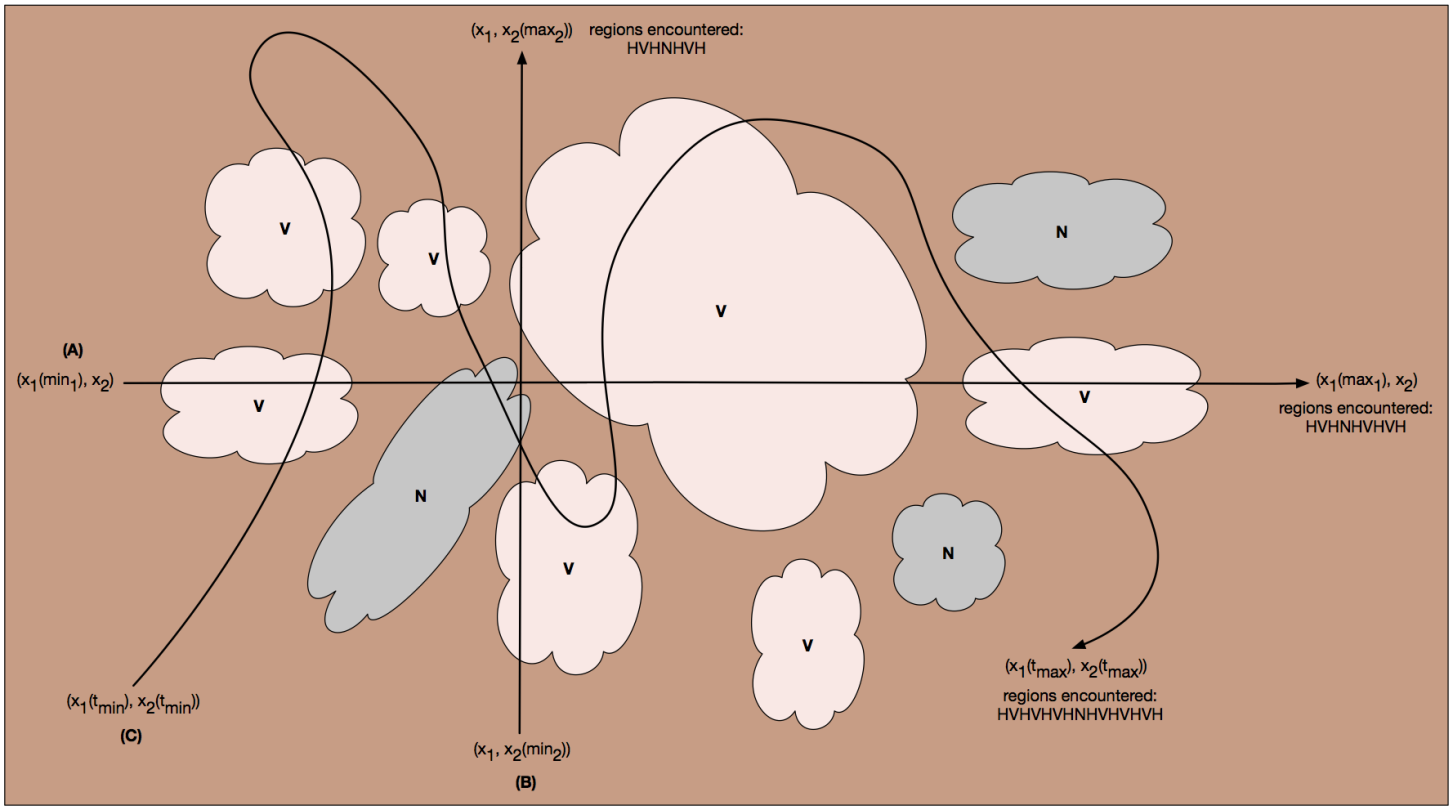
Verifying axiom *A1*. One way to verify that viable (*V*, tan) and necrotic (*N*, gray) regions are nowhere contiguous (i.e., they are everywhere separated by a hypoxic region (*H*, brown) is to follow arbitrary trajectories in the 2D or 3D space, each of which represents a spatiotemporal logical proposition, any number of whose results can be conjoined to obtain a system-wide propositional truth value. (A) The trajectory obtained by holding *x*_2_ fixed at some arbitrary value and allowing *x*_1_ to vary across an arbitrary extent, from some *min*_1_ to some *max*_1_. (B) The trajectory obtained by holding *x*_1_ fixed at some arbitrary value and allowing *x*_2_ to vary across an arbitrary extent, from some *min*_2_ to some *max*_2_. (C) A curvilinear trajectory, parameterized here by some arbitrary *t*, extending from some *t*_*min*_ to some *t*_*max*_.

Suppose we are in a 2D universe. From axiom *A1* above, we can immediately write our first proposition term. If we hold *x*_2_ fixed at any arbitrary value and test along *x*_1_ from an arbitrary *min*_1_ to an arbitrary *max*_1_ value, *x*_1_(*min*_1_)→*x*_1_(*max*_1_), then we expect to encounter tissue types in the following pattern: {*V*, *N*}→*H* → {*V*, *N*}→*H* → …., that is, any contiguous *V* or *N* region is separated by a contiguous *H* region (see trajectory (A) in Fig 7). This is equivalent to axiom *A1*, which states that *V* and *N* are invalid neighbors, their regions cannot abut. Suppose we have a primitive state variable function *T*: (*x*_1_, *x*_2_)→{*H*, *V*, *N*} that given a coordinate (*x*_1_, *x*_2_) returns the tissue type at that coordinate, namely *H*, *V*, or *N*. In terms of our spatiotemporal logic, we can implement a verification of axiom *A1* with the following proposition term:

$$\begin{array}{c} \neg [\left(T\left(x_{1},x_{2}\right)=N\right)U^{x_{1},max_{1}-min_{1}}\left(T\left(x_{1},x_{2}\right)=V\right)]\\ \wedge \\ \neg [\left(T\left(x_{1},x_{2}\right)=V\right)U^{x_{1},max_{1}-min_{1}}\left(T\left(x_{1},x_{2}\right)=N\right)]. \end{array}$$ (1)

Because the valid spatiotemporal grammatical transformation rules apply symmetrically, we can write the analogous propositional term for holding *x*_1_ fixed at some arbitrary value and testing along *x*_2_ from an arbitrary *min*_2_ to an arbitrary *max*_2_ value, *x*_2_(*min*_2_)→*x*_2_(*max*_2_) (see trajectory (B) in Fig 7):

$$\begin{array}{c} \neg [\left(T\left(x_{1},x_{2}\right)=N\right)U^{x_{2},max_{2}-min_{2}}\left(T\left(x_{1},x_{2}\right)=V\right)]\\ \wedge \\ \neg [\left(T\left(x_{1},x_{2}\right)=V\right)U^{x_{2},max_{2}-min_{2}}\left(T\left(x_{1},x_{2}\right)=N\right)]. \end{array}$$ (2)

In fact, we can define any curvilinear path through our space, (*x*_1_(*t*_*min*_), *x*_2_(*t*_*min*_)) → (*x*_1_(*t*_*max*_), *x*_2_(*t*_*max*_)), in this case parametric in some *t* (see trajectory (C) in Fig 7), and write an analogous propositional term. Another systematic approach to verifying axiom *A1* (or any testable pattern) would be to use some combination of the above to implement a space-filling curve in 2D or 3D, for example a Peano or Hilbert curve.

**Fig 8:**
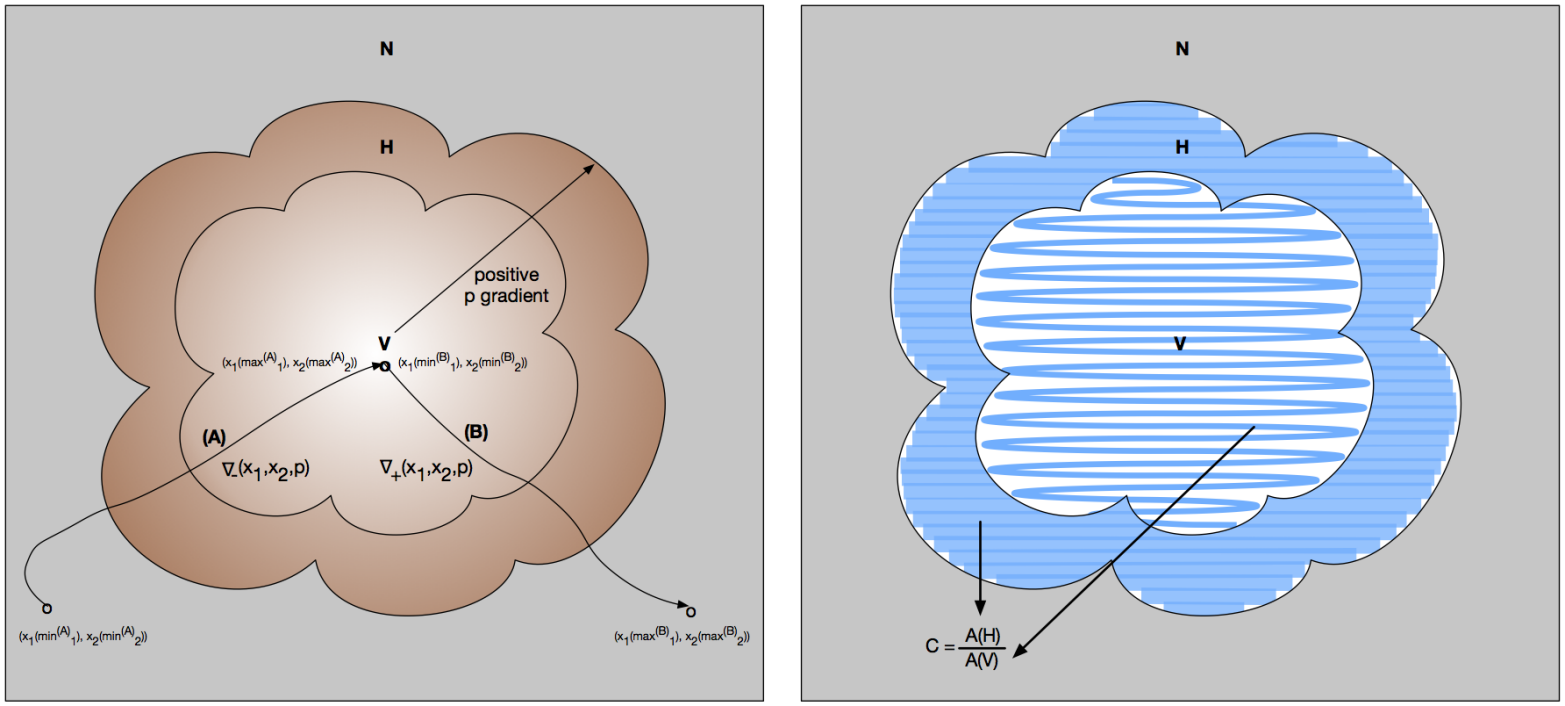
Hypoxia gradients and segmented tissue areas. Left panel: verifying the hypoxia gradient image feature against empirically measured and statistically bounded values. (A) The trajectory obtained by starting at $\left(x_{1}\left(min_{1}^{\left(A\right)}\right),x_{2}\left(min_{2}^{\left(A\right)}\right)\right)$ in a necrotic (*N*) region, locating a hypoxic (*H*) region, and then descending the hypoxia (*p* = anti-pimonidazole) gradient into a viable (*V*) region to reach the vessel centroid at $\left(x_{1}\left(max_{1}^{\left(A\right)}\right),x_{2}\left(max_{2}^{\left(A\right)}\right)\right)$, using the ∇_−_(*x*_1_, *x*_2_, *p*) gradient following function. (B) The trajectory obtained by starting at the vessel centroid at $\left(x_{1}\left(min_{1}^{\left(B\right)}\right),x_{2}\left(min_{2}^{\left(B\right)}\right)\right)$ in the viable (*V*) region, then ascending the hypoxia (*p* = anti-pimonidazole) gradient into a hypoxic (*H*) region until reaching a necrotic (*N*) region at $\left(x_{1}\left(max_{1}^{\left(B\right)}\right),x_{2}\left(max_{2}^{\left(B\right)}\right)\right)$, using the ∇_+_(*x*_1_, *x*_2_, *p*) gradient following function. Right panel: verifying the viable-to-hypoxic area ratio ($C=\frac{A(V)}{A(H)}$) image feature against empirically measured and statistically bounded values.

Next we turn to our image feature for a two-segment gradient. Suppose we have a primitive state variable function ∇_+_: (*x*_1_, *x*_2_, *p*)→(*δ*_*x*_1__, *δ*_*x*_2__, *δρ*_*p*_) that given a coordinate (*x*_1_, *x*_2_) and a particle type *p* returns two items: the coordinate (*δ*_*x*_1__, *δ*_*x*_2__) adjacent to (*x*_1_, *x*_2_) that has the greatest concentration of *p*, and *δ*_*ρ*_*p*__, the measure of that greatest concentration of *p*. So ∇_+_ is a function that performs gradient ascent. For completeness, suppose too that we have the analogous function ∇_−_ to perform gradient descent. Thus by starting at some vessel centroid $\left(x_{1}\left(min_{1}^{\left(B\right)}\right),x_{2}\left(min_{2}^{\left(B\right)}\right)\right)$ in viable region *V*, and extending along a contour iteratively specified by ∇_+_, we will eventually encounter the boundary of the hypoxic region *H*, followed by the boundary of the necrotic region *N*, eventually ending at $\left(x_{1}\left(max_{1}^{\left(B\right)}\right),x_{2}\left(max_{2}^{\left(B\right)}\right)\right)$, all the while ascending the gradient of *p* (in this case, anti-pimonidazole) concentration (see trajectory (B) in the left panel of Fig 8). This gives our next propositional term:

$$\begin{array}{c} \left(T\left(x_{1},x_{2}\right)=V\right)\\ U^{\left(x_{1},x_{2}\right)\leftarrow \nabla_{+}\left(x_{1},x_{2},p\right),\left(max_{1}^{\left(B\right)}-min_{1}^{\left(B\right)},max_{2}^{\left(B\right)}-min_{2}^{\left(B\right)}\right)}\\ \left(T\left(x_{1},x_{2}\right)=H\right)\\ U^{\left(x_{1},x_{2}\right)\leftarrow \nabla_{+}\left(x_{1},x_{2},p\right),\left(max_{1}^{\left(B\right)}-min_{1}^{\left(B\right)},max_{2}^{\left(B\right)}-min_{2}^{\left(B\right)}\right)}\\ \left(T\left(x_{1},x_{2}\right)=N\right)\\ \wedge \\ \left(F^{\left(x_{1},x_{2}\right)\leftarrow \nabla_{+}\left(x_{1},x_{2},p\right),172+83}\left(\nabla_{+}\left(x_{1},x_{2},p\right)=-0.21\pm 0.19\right)\right)\\ U^{\left(x_{1},x_{2}\right)\leftarrow \nabla_{+}\left(x_{1},x_{2},p\right),\left(172+83+267+126\right)}\\ (F^{\left(x_{1},x_{2}\right)\leftarrow \nabla_{+}\left(x_{1},x_{2},p\right),267+126}\left(\nabla_{+}\left(x_{1},x_{2},p\right)=-0.06\pm 0.03\right)), \end{array}$$ (3)

which in English means “Along the total arc length of trajectory (B), we are in viable tissue until we are in hypoxic tissue until we are in necrotic tissue. And for the arc length of trajectory (B), we verify the gradient trajectory characterized by our experimental results in the following manner: for segment one, we follow a mean gradient slope of -0.21 (bounded from above and below by standard deviation 0.19) for an arc length bounded from above by the mean length of segment one, 172, plus its standard deviation, 83, until we reach segment two; then for segment two, we follow a mean gradient slope of -0.06 (bounded from above and below by standard deviation 0.03) for an arc length bounded from above by the mean length of segment two, 267, plus its standard deviation, 126.” The gradient segment lengths, length bounds, slopes, and slope bounds are given in Table 6, and all measurements are in pixels. This is merely one provisional gradient term, and not intended to represent a comprehensive gradient characterization in spatiotemporal logical terms. Note too that we could write an analogous propositional term by reordering the clauses to reflect the opposite order of encounter, and by using negated slopes and the gradient descent function ∇_−_ (see trajectory (A) in the left panel of Fig 8).

Now we look at our segmentation image feature, and the derived ratio of cell types that can be represented by its values. Suppose we have a primitive state function C:∅→Q that gives the current ratio of hypoxic to viable cells in some spatially bounded area (see the right panel of Fig 8). Then given the results from our segmentation experiment below (Table 1), namely for the mean *H*:*V* ratio, we have the following temporal term, with respect to *t*_0_:

$$G^{t,\tau }\left(C=0.36\pm 0.24\right),$$ (4)

where *τ* is an upper bound on the time (e.g., the run time of a simulation that seeks to detect hypoxia).

If we assume the four propositional terms above are true for time bounded by *τ*, then our final (but partial) spatiotemporal logical proposition characterizing hypoxia is given by:

$$\begin{array}{c} G^{t,\tau }\\ (\\ \neg [\left(T\left(x_{1},x_{2}\right)=N\right)U^{x_{1},max_{1}-min_{1}}\left(T\left(x_{1},x_{2}\right)=V\right)]\;\;\;\;\\ \wedge \;\;\;\;\\ \neg [\left(T\left(x_{1},x_{2}\right)=V\right)U^{x_{1},max_{1}-min_{1}}\left(T\left(x_{1},x_{2}\right)=N\right)]\;\;\;\;\\ \wedge \\ \neg [\left(T\left(x_{1},x_{2}\right)=N\right)U^{x_{2},max_{2}-min_{2}}\left(T\left(x_{1},x_{2}\right)=V\right)]\;\;\;\;\\ \wedge \;\;\;\;\\ \neg [\left(T\left(x_{1},x_{2}\right)=V\right)U^{x_{2},max_{2}-min_{2}}\left(T\left(x_{1},x_{2}\right)=N\right)]\;\;\;\;\\ \wedge \\ (T\left(x_{1},x_{2}\right)=V)\;\;\;\;\\ U^{\left(x_{1},x_{2}\right)\leftarrow \nabla_{+}\left(x_{1},x_{2},p\right),\left(max_{1}^{\left(B\right)}-min_{1}^{\left(B\right)},max_{2}^{\left(B\right)}-min_{2}^{\left(B\right)}\right)}\;\;\;\;\\ (T\left(x_{1},x_{2}\right)=H)\;\;\;\;\\ U^{\left(x_{1},x_{2}\right)\leftarrow \nabla_{+}\left(x_{1},x_{2},p\right),\left(max_{1}^{\left(B\right)}-min_{1}^{\left(B\right)},max_{2}^{\left(B\right)}-min_{2}^{\left(B\right)}\right)}\;\;\;\;\\ (T\left(x_{1},x_{2}\right)=N)\;\;\;\;\\ \wedge \;\;\;\;\\ (F^{\left(x_{1},x_{2}\right)\leftarrow \nabla_{+}\left(x_{1},x_{2},p\right),172+83}\left(\nabla_{+}\left(x_{1},x_{2},p\right)=-0.21\pm 0.19\right))\;\;\;\;\\ U^{\left(x_{1},x_{2}\right)\leftarrow \nabla_{+}\left(x_{1},x_{2},p\right),\left(172+83+267+126\right)}\;\;\;\;\\ (F^{\left(x_{1},x_{2}\right)\leftarrow \nabla_{+}\left(x_{1},x_{2},p\right),267+126}\left(\nabla_{+}\left(x_{1},x_{2},p\right)=-0.06\pm 0.03\right))\;\;\;\;\\ \wedge \\ (C=0.36\pm 0.24)\;\;\;\;\\ ), \end{array}$$ (5)

where fixed *x*_1_, fixed *x*_2_, $min_{1},max_{1},min_{2},max_{2},min_{1}^{\left(B\right)},max_{1}^{\left(B\right)},min_{2}^{\left(B\right)}$, and $max_{2}^{\left(B\right)}$ are allowed to vary arbitrarily (or programmatically, for example in a simulator that seeks to detect hypoxia in some systematic search pattern).

## Conclusion

We propose an approach to characterizing chronic tumor hypoxia by utilizing the rich content of tumor histology images of different kinds. We discovered that two features in particular give low-variance measures of chronic tumor hypoxia: intensity sampling that extends radially away from approximated blood vessel centroids, and multithresholding to segment tumor tissue into normal, hypoxic, and necrotic regions. From these features we derived a spatiotemporal logical expression whose truth value depends on its predicate clauses that are grounded in this histological evidence. As an alternative to the spatiotemporal logical formulation, we also proposed a way to formulate a linear regression function that uses all of the image features to learn what chronic hypoxia looks like, and then gives a quantitative similarity score once it is trained on a set of histology images. One can extend these logical and functional characterizations of chronic tumor hypoxia to incorporate any number of quantitative features. The functional characterization also provides a means to parse systematic error and model error, quantify them, and control for them, leading to progressively less bias in solving the detection problem. Our techniques could be used to help detect chronic tumor hypoxia in a clinical setting, or in a research setting, as part of a simulation exploring causes of chronic tumor hypoxia.

Our simplified system is represented by histology image data from tumor sites selected to be near blood vessels, and at length scales small enough to observe chronic hypoxia. We acknowledge our simplified system does not represent the full complexity of tumor vascularity and hypoxia. Whole-tumor measurement and diagnosis, while of great value, is beyond the scope of this study. Two benefits of our computational method of characterization and detection are that it is automatable and scalable, and thus we envision our method could become the foundation for investigating more complex situations that represent a more holistic view of tumor hypoxia. For example, a proper sampling of chronic hypoxia sites could be used to assemble a whole-tumor estimation of global chronic hypoxia distribution. This type of bottom-up modeling out of many partial measurements could be used to produce a mathematical model that has some generality over many tumors and tumor types; given a novel tumor, such a model might be able to predict the tumor’s global chronic hypoxia distribution. We leave this investigation for a future study.

In light of these research goals, we pose two caveats. First, the basis of our characterization method is image processing of tumor slice histology images. While these data give ample structural features to quantify, they do not contain the dynamic complexity of living tumor tissue. Thus, the resulting characterizations we develop from histology alone cannot properly capture tumor heterogeneity, which makes it difficult to generalize a given characterization of chromic tumor hypoxia to novel tumor sites. Second, though we did investigate a variety of images for our study, we stratified them, selecting the subset which best fit our purpose of quantifying the gradient near vessels in the tumor. These images largely show “tumor cords” widely separated by necrosis. While this is an ideal setting for our analysis of the hypoxia gradient feature, it may exist in a minority of tumors, which researchers have suspected since the first landmark study of “tumor cords” [4]. If one wishes to use tumor histology images as evidence of chronic tumor hypoxia, then a robust quantitative characterization will require a larger study, covering a large variety of tumors and a broader selection of sites within each tumor.

## Supporting Information

S1 Fig Image preprocessing. Our canonical image as an 8-bit grayscale image (top) and after iterative smoothing (bottom).
(TIF)

S2 Fig Preliminary qualitative analysis of image intensity. Our smoothed canonical image plotted as a mesh (top) and a contour (bottom). Both show a qualitative tri-level partitioning of image intensity.
(TIF)

S3 Fig Preliminary histogram analysis of image intensity. When we examine all of the pixels of our smoothed canonical image (upper left), we see a clear bimodal distribution in the intensity histogram (upper right). Yet, when we select a sub-image where we see roughly equal proportions of the three distinct tissue types (lower left), a trimodal distribution appears in the intensity histogram (lower right).
(TIF)

S4 Fig Tissue types by manual segmentation of our smoothed canonical image. Hypoxic tissue, as defined by the intensity interval [0, 156] (top). Viable tissue, as defined by the intensity interval [157, 175] (middle). Note the false-positive outer contours around the hypoxic tissue, and the false-negative inner backbone areas where there are collagen deposits. Necrotic tissue, as defined by the intensity interval [176, 255] (bottom). Note the false positive areas where collagen forms an inner backbone that partitions the viable tissue.
(TIF)

S5 Fig Quad-Tree decomposition. A quad-tree decomposition of our canonical image, where the criterion for decomposition of a given frame is a sufficiently high variation among the frame’s pixels’ intensity values.
(TIF)

S6 FigLoci of eight-bundle hypoxia gradients.Circle sectors (red) defined by the *r*_*m*_ found by our Intensity-Sample-Ray-Bundles algorithm for each bundle of each of the three centers we specified, corresponding to vessel locations in the registered H&E image. Here we show *m* = 8 sectors ($\frac{\pi }{4}$ radians per sector) for each center. Sectors are labeled with red numbers, counterclockwise, just outside of the red sector contour.(TIF)

S7 Fig Histology image registration. Registering an H&E image (not shown) to a *Z*-stack-adjacent anti-pimonidazole image (10 *μ*m away). The three blue vectors denote the displacements of the three gradient centers. Each blue vector is labeled with *P*_*i*_ at the head (center position *i* in the anti-pimonidazole image) and *H*_*i*_ at the tail (the corresponding center position *i* in the H&E image). The vector lengths (in pixels) are labeled, as are the vector angles (in radians), measured relative to their respective dotted blue horizontal lines.
(TIF)

S8 FigQuad-Tree-Ply-Stats results.How the Ply-Stats-Quad-Tree algorithm dissects images according to the property of CV in intensity level of a given frame’s pixels: the mean window size profile across the *total* (left three panels, *n* = 66), *high* (middle three panels, *n* = 36), and *low* (right three panels, *n* = 30) sets of images. In each set of three panels, the first, second, and third panels show mean histograms for *τ* ∈ {0.1, 0.5, 0.9}, respectively. The horizontal axis indicates ply depth, or frame size as computed by $\frac{x\_dim}{2^{i}}\times \frac{y\_dim}{2^{i}}$, where *i* ∈ [0, 12] is the ply depth, and *x*_*dim* and *y*_*dim* are the *x* and *y* dimensions of the whole image, respectively. The vertical axis indicates the mean count of search tree leaves at ply depth *i*. Error bars show standard deviation.(TIF)

S9 Fig Canonical EPC surve results. (Top) The mean EPC curve over the *total* set of images (left, *n* = 66), the *high* concentration anti-pimonidazole images (middle, *n* = 36), and the *low* concentration anti-pimonidazole images (right, *n* = 30). Segmented least-squares fits to these curves are given below. The horizontal axis indicates the intensity level threshold *τ* ∈ [1, 255] applied to the image prior to computing *χ*. The vertical axis indicates the value of *χ* computed for each *τ*. (Bottom) The segmented least-squares fits to the mean EPC curves given above. The horizontal axis indicates the intensity level threshold *τ* ∈ [1, 255] applied to the image prior to computing *χ*. The vertical axis indicates the value of *χ* computed for each *τ*.
(TIF)

## References

[1] Höckel M, Schlenger K, Aral B, Mitze M, Schäffer U, Vaupel P. Association between tumor hypoxia and malignant progression in advanced cancer of the uterine cervix. Cancer Research. 1996;56:4509–4515. 8813149

[2] Vaupel P, Mayer A. Hypoxia in cancer: significance and impact on clinical outcome. Cancer Metastasis Review. 2007;26:225–239. 10.1007/s10555-007-9055-1 17440684

[3] Green DR. Means to an End: Apoptosis and Other Cell Death Mechanisms. Cold Spring Harbor Laboratory Press; 2011.

[4] Thomlinson RH, Gray LH. The histological structure of some human lung cancers and the possible implications for radio-therapy. British Journal of Cancer. 1955;9(4):539–549. 10.1038/bjc.1955.55 13304213 PMC2073776

[5] Osinsky S, Bubnovskaya L, Ganusevich I, Kovelskaya A, Gumenyuk L, Olijnichenko G, et al Hypoxia, tumour-associated macrophages, microvessel density, VEGF and matrix metalloproteinases in human gastric cancer: interaction and impact on survival. Clinical and Translational Oncology. 2011;13(2):133–138. 10.1007/s12094-011-0630-0 21324802

[6] Giaccia AJ, Schipani E. Role of carcinoma-associated fibroblasts and hypoxia in tumor progression. Current Topics in Microbiology and Immunology. 2010;345:31–45. 20517716 10.1007/82_2010_73

[7] Cirri P, Chiarugi P. Cancer associated fibroblasts: the dark side of the coin. American Journal of Cancer Research. 2011;1(4):482–497. 21984967 PMC3186047

[8] Simon MC, editor. Diverse Effects of Hypoxia on Tumor Progression. vol. 345 Springer; 2010.

[9] Cell. Oxygen sensing in cancer and metabolism; 2013.

[10] Cárdenas-Navia LI, Mace D, Richardson RA, Wilson DF, Shan S, Dewhirst MW. The pervasive presence of fluctuating oxygenation in tumors. Cancer Research. 2008;68(14):5812–5819. 10.1158/0008-5472.CAN-07-6387 18632635

[11] Tannock IF. Population kinetics of carcinoma cells, capillary endothelial cells, and fibroblasts in a transplanted mouse mammary tumor. Cancer Research. 1970;30(10):2470–2476. 4097429

[12] Krogh A. The rate of diffusion of gases through animal tissues, with some remarks on the coefficient of invasion. Journal of Physiology. 1919;52:391–408. 10.1113/jphysiol.1919.sp001838 16993404 PMC1402717

[13] Krogh A. The number and distribution of capillaries in muscles with calculations of the oxygen pressure head necessary for supplying the tissue. Journal of Physiology. 1919;52:409–415. 10.1113/jphysiol.1919.sp001839 16993405 PMC1402716

[14] Krogh A. The supply of oxygen to the tissues and the regulation of the capillary circulation. Journal of Physiology. 1919;52:457–474. 10.1113/jphysiol.1919.sp001844 16993410 PMC1402718

[15] Weibel ER. The structural conditions for oxygen supply to muscle cells: the Krogh cylinder model. Journal of Experimental Biology. 2013;216:4135–4137. 10.1242/jeb.076497 24172885

[16] Goldman D. Theoretical models of microvasculature oxygen transport to tissue. Microcirculation. 2008;15(8):795–811. 10.1080/10739680801938289 18608981 PMC3057578

[17] Kang K, Bruley D, Bicher H. A computer simulation of simultaneous heat and oxygen transport during heterogeneous three dimensional tumor hyperthermia. Advances in Experimental Medicine and Biology. 1988;222:747–756. 10.1007/978-1-4615-9510-6_92 3364302

[18] Kavanagh B, Secomb T, Hsu R, Lin P, Venitz J, Dewhirst M. A theoretical model for the effects of reduced hemoglobin-oxygen affinity on tumor oxygenation. International Journal of Radiation Oncology, Biology, Physics. 2002;53:172–179. 10.1016/S0360-3016(02)02740-2 12007957

[19] JP JK, Brizel D, Dewhirst M. A mathematical model of tumor oxygen and glucose mass transport and metabolism with complex reaction kinetics. Radiation Research. 2003;59:336–344. 10.1667/0033-7587(2003)159[0336:ammoto]2.0.co;2 12600236

[20] Secomb T, Hsu R, Dewhirst M, Klitzman B, Gross J. Analysis of oxygen transport to tumor tissue by microvascular networks. International Journal of Radiation Oncology, Biology, Physics. 1993;25(3):481–489. 10.1016/0360-3016(93)90070-C 8436527

[21] Helmlinger G, Yuan F, Dellian M, Jain RK. Interstitial pH and *pO*_2_ gradients in solid tumors in vivo: high-resolution measurements reveal a lack of correlation. Nature Medicine. 1997;3(2):177–182. 10.1038/nm0297-177 9018236

[22] Wang CW, Fennell D, Paul I, Savage K, Hamilton P. Robust Automated Tumour Segmentation on Histological and Immunohistochemical Tissue Images. PLoS ONE. 2011;6(2:e15818). 10.1371/journal.pone.0015818 21386898 PMC3046129

[23] Otsu N. A threshold selection method from gray-level histograms. IEEE Transactions on Systems, Man, and Cybernetics. 1979;9(1):62–66. 10.1109/TSMC.1979.4310076

[24] Jha SK, Clarke EM, Langmead CJ, Legay A, Platzer A, Zuliani P. A Bayesian approach to model checking biological systems In: Degano P, Gorrieri R, editors. Computational Methods in Systems Biology 2009 (LNBI 5688). Springer-Verlag Berlin Heidelberg; 2009 p. 218–234.

[25] Zuliani P, Platzer A, Clarke EM. Bayesian statistical model checking with application to Stateflow/Simulink verification. In: International Conference on Hybrid Systems: Computation and Control 2010; 2010. p. 243–252.

[26] Gong H, Zuliani P, Komuravelli A, Faeder J, Clarke EM. Computational modeling and verification of signaling pathways in cancer. In: Horimoto K, Nakatsu M, Popov N, editors. International Conference on Algebraic and Numeric Biology 2011 (LNCS 6479). Springer-Verlag Berlin Heidelberg; 2010. p. 117–135.

[27] Gong, H, Zuliani, P, Komuravelli, A, Faeder, J, Clarke, EM. Analysis and verification of the HMGB1 signaling pathway. In: Asia Pacific Bioinformatics (APBioNet) International Conference on Bioinformatics 2010 (InCoB2010). BMC Bioinformatics 2010, 11(Suppl 7):S10; 2010. p. 1–13. 10.1186/1471-2105-11-S7-S10 PMC2957678 21106117

[28] Grosu R, Smolka SA, Corradini F, Wasilewska A, Entcheva E, Bartocci E. Learning and detecting emergent behavior in networks of cardiac myocytes. Communications of the ACM. 2009;52(3):97–105. 10.1145/1467247.1467271

[29] Sundstrom A, Cirrone S, Paxia S, Hsueh C, Kjolby R, Gimzewski JK, et al Image analysis and length estimation of biomolecules using AFM. IEEE Transactions on Information Technology in Biomedicine. 2012;16(6):1200–1207. 10.1109/TITB.2012.2206819 22759526 PMC4207372

[30] Kleinberg J, Éva Tardos. 6. In: Algorithm Design. Addison Wesley; 2006. p. 261–266.

[31] Michielsen K, Raedt HD. Integral-geometry morphological image analysis. Physics Reports. 2001;347:461–538. 10.1016/S0370-1573(00)00106-X

[32] Legland D, Kiêu K, Devaux MF. Computation of Minkowski measures on 2D and 3D binary images. Image Analysis & Stereology. 2007;26:83–92. 10.5566/ias.v26.p83-92

[33] Guderlei R, Klenk S, Mayer J, Schmidt V, Spodarev E. Algorithms for the computation of the Minkowski functionals of deterministic and random polyconvex sets. Image and Vision Computing. 2007;25(4):464–474. 10.1016/j.imavis.2006.07.019

[34] Räth C, Monetti R, Bauer J, Sidorenko I, Müller D, Matsuura M, et al Strength through structure: visualization and local assessment of the trabecular bone structure. New Journal of Physics. 2008;10:1–18.

[35] Roque WL, de Souza ACA, Berbieri DX. The Euler-Poincaré characteristic applied to identify low bone density from vertebral tomographic images. Brazilian Journal of Rheumatology. 2009;49(2):146–152.

[36] Hutterer S, Zauner G, Huml M, Silye R, Schilcher K. Data mining techniques for AFM-based tumor classification. In: 2012 IEEE Symposium on Computational Intelligence in Bioinformatics and Computational Biology (CIBCB); 2012. p. 105–111.

[37] Sen A, Srivastava M. 12. In: Regression Analysis: Theory, Methods, and Applications. Springer; 1990 p. 253–262.

[38] Beaton AE, Tukey JW. The fitting of power series, meaning polynomials, illustrated on band-spectroscopic data. Technometrics. 1974;16(2):147–185. 10.1080/00401706.1974.10489171

[39] James W, Stein C. Estimation with quadratic loss. In: Proceedings of the Berkeley Symposium on Mathematical Statistical Probability; 1961. p. 316–379.

