# Supplementary figures and images for: Histological Image Processing Features Induce a Quantitative Characterization of Chronic Tumor Hypoxia
Source: PLoS One. 2016 Apr 19;11(4):e0153623. doi: 10.1371/journal.pone.0153623 (PMC4836667; doi:10.1371/journal.pone.0153623)

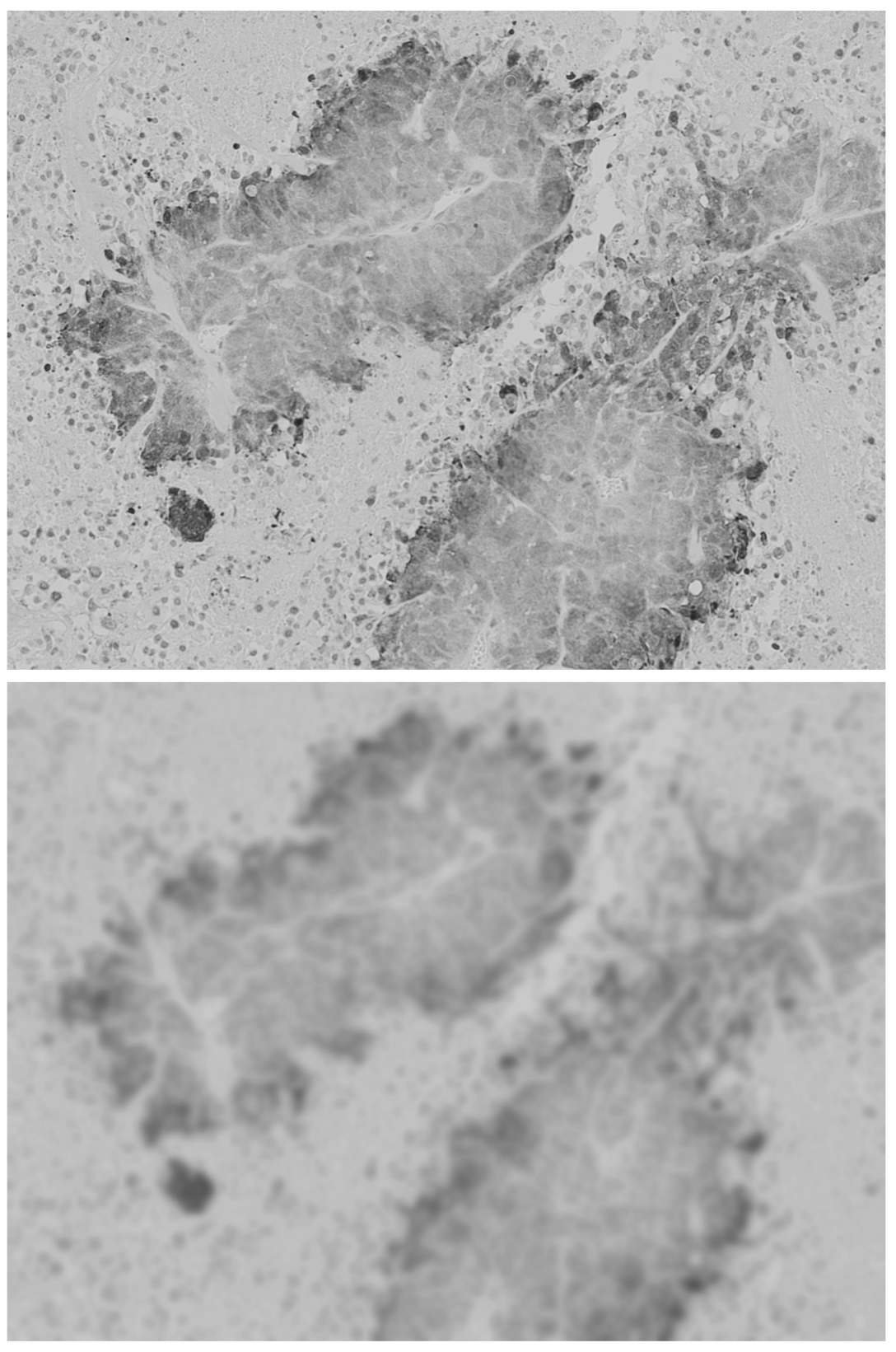

Supplement: S1 Fig — Our canonical image as an 8-bit grayscale image (top) and after iterative smoothing (bottom). (TIF) [file pone.0153623.s001.tif]

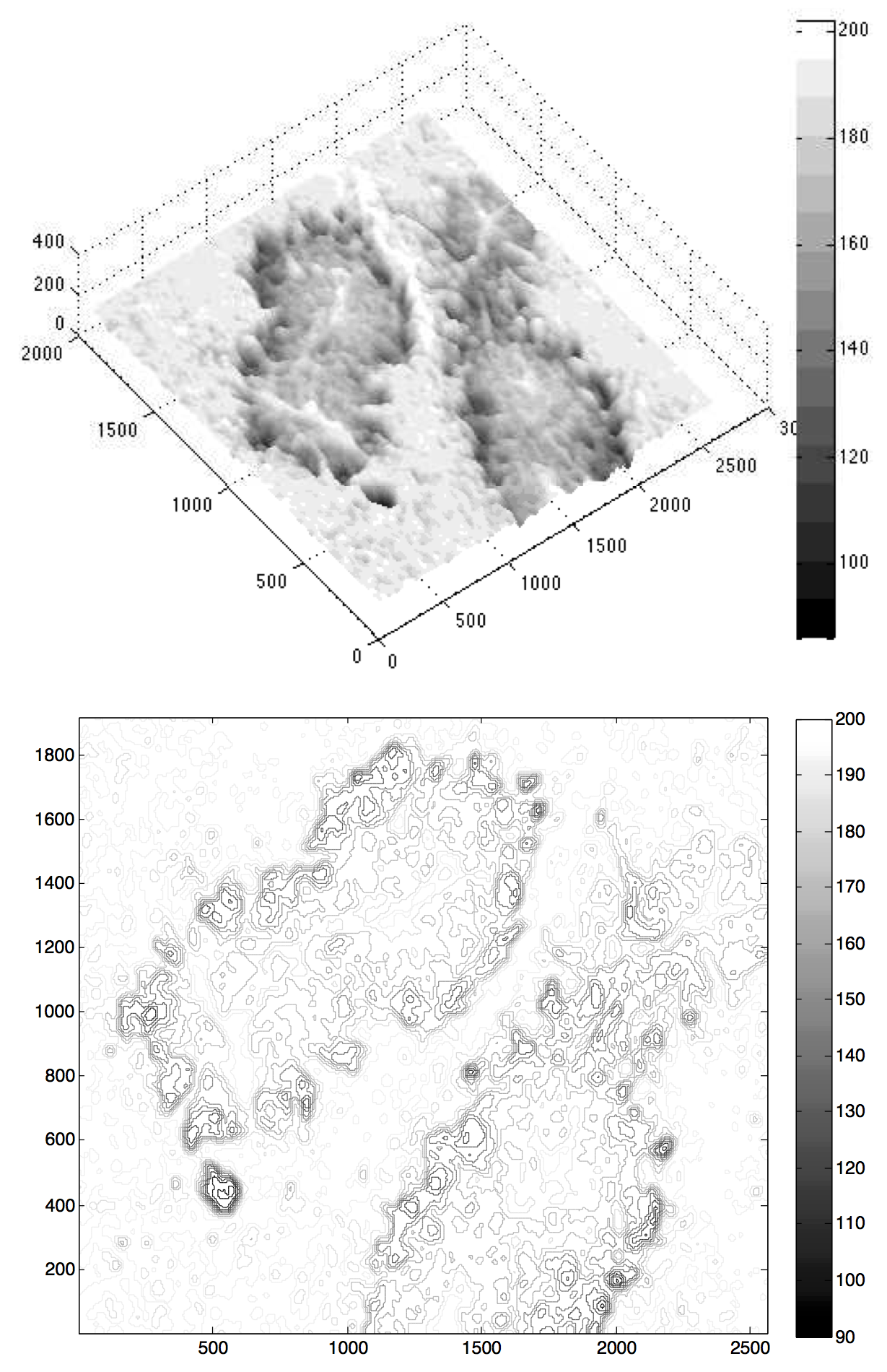

Supplement: S2 Fig — Our smoothed canonical image plotted as a mesh (top) and a contour (bottom). Both show a qualitative tri-level partitioning of image intensity. (TIF) [file pone.0153623.s002.tif]

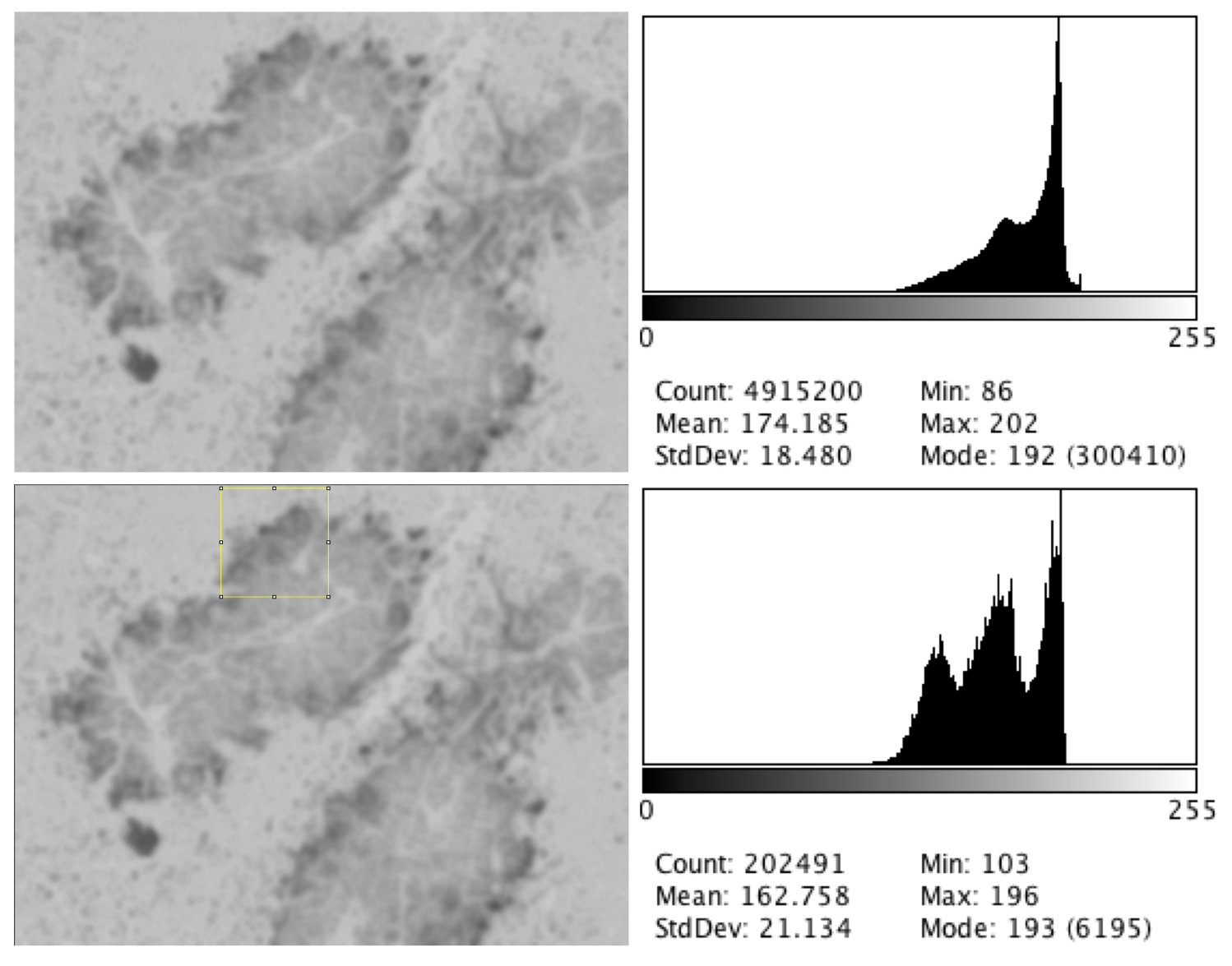

Supplement: S3 Fig — When we examine all of the pixels of our smoothed canonical image (upper left), we see a clear bimodal distribution in the intensity histogram (upper right). Yet, when we select a sub-image where we see roughly equal proportions of the three distinct tissue types (lower left), a trimodal distribution appears in the intensity histogram (lower right). (TIF) [file pone.0153623.s003.tif]

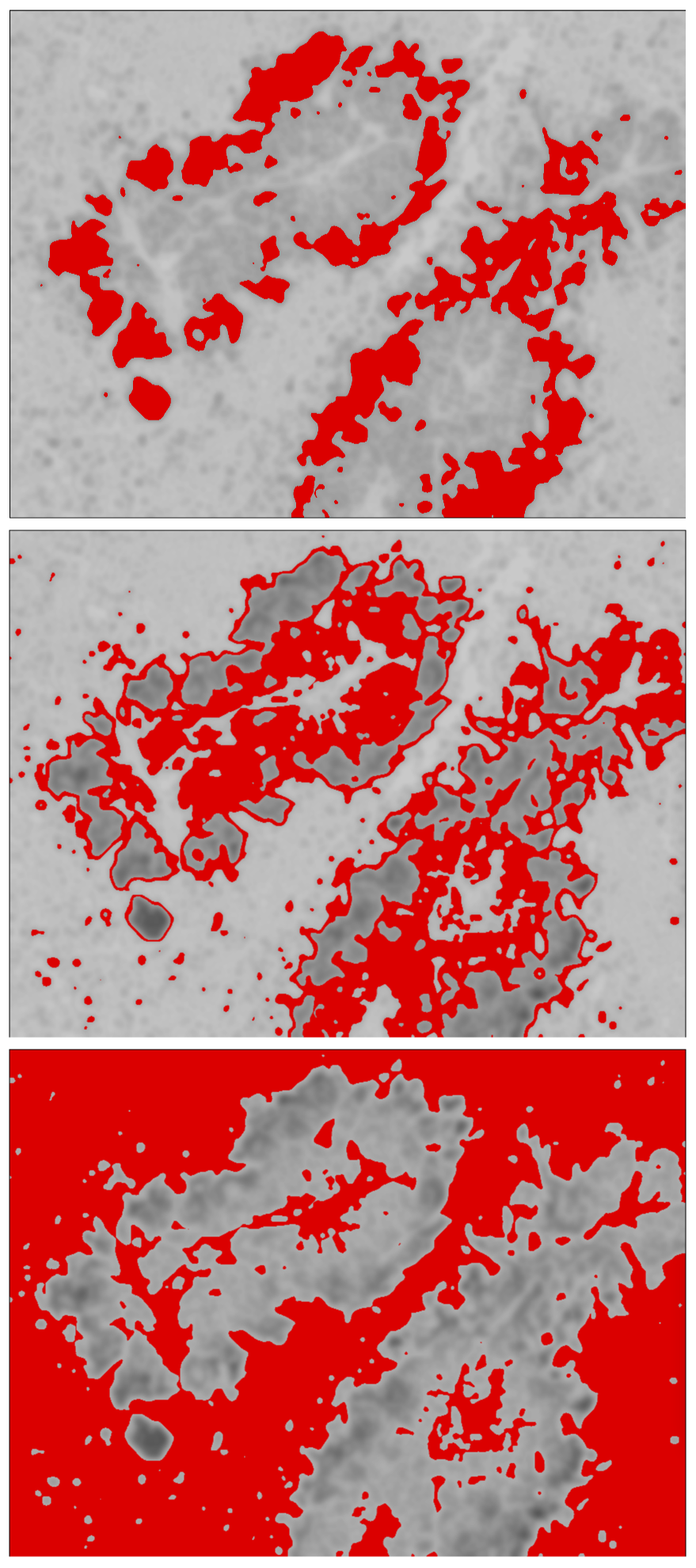

Supplement: S4 Fig — Hypoxic tissue, as defined by the intensity interval [0, 156] (top). Viable tissue, as defined by the intensity interval [157, 175] (middle). Note the false-positive outer contours around the hypoxic tissue, and the false-negative inner backbone areas where there are collagen deposits. Necrotic tissue, as defined by the intensity interval [176, 255] (bottom). Note the false positive areas where collagen forms an inner backbone that partitions the viable tissue. (TIF) [file pone.0153623.s004.tif]

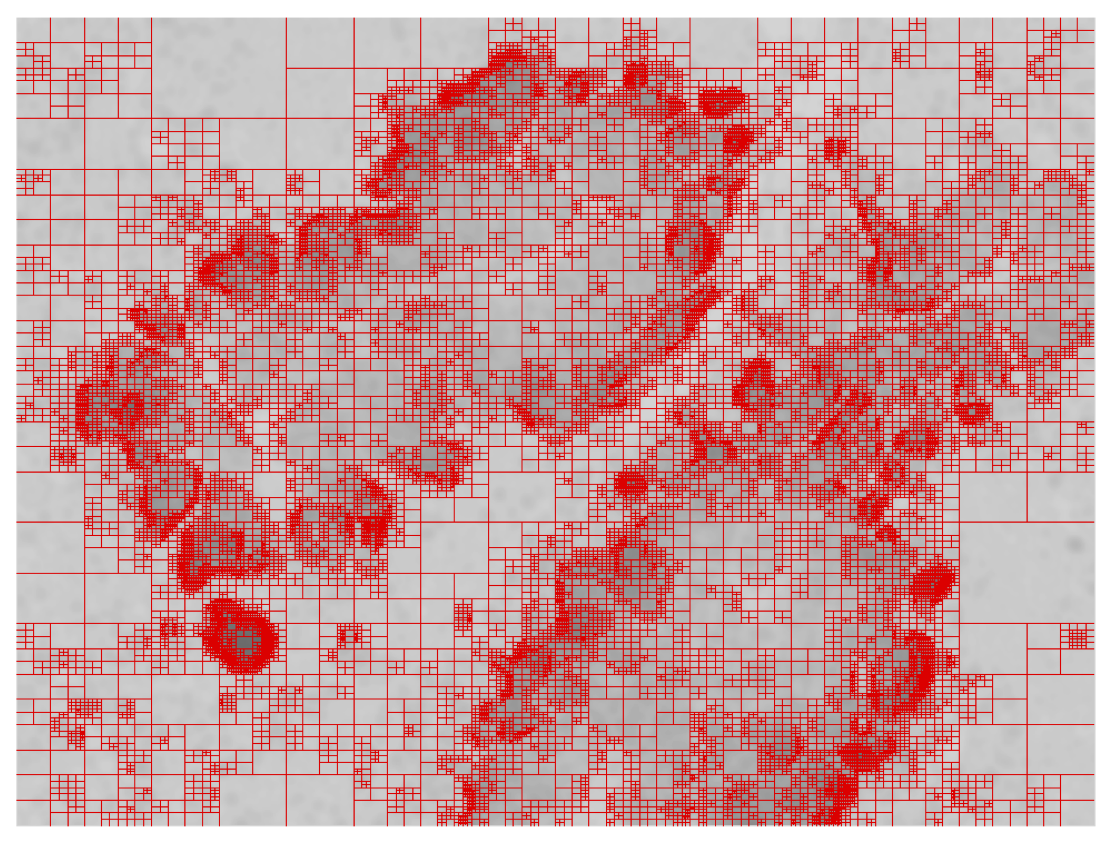

Supplement: S5 Fig — A quad-tree decomposition of our canonical image, where the criterion for decomposition of a given frame is a sufficiently high variation among the frame’s pixels’ intensity values. (TIF) [file pone.0153623.s005.tif]

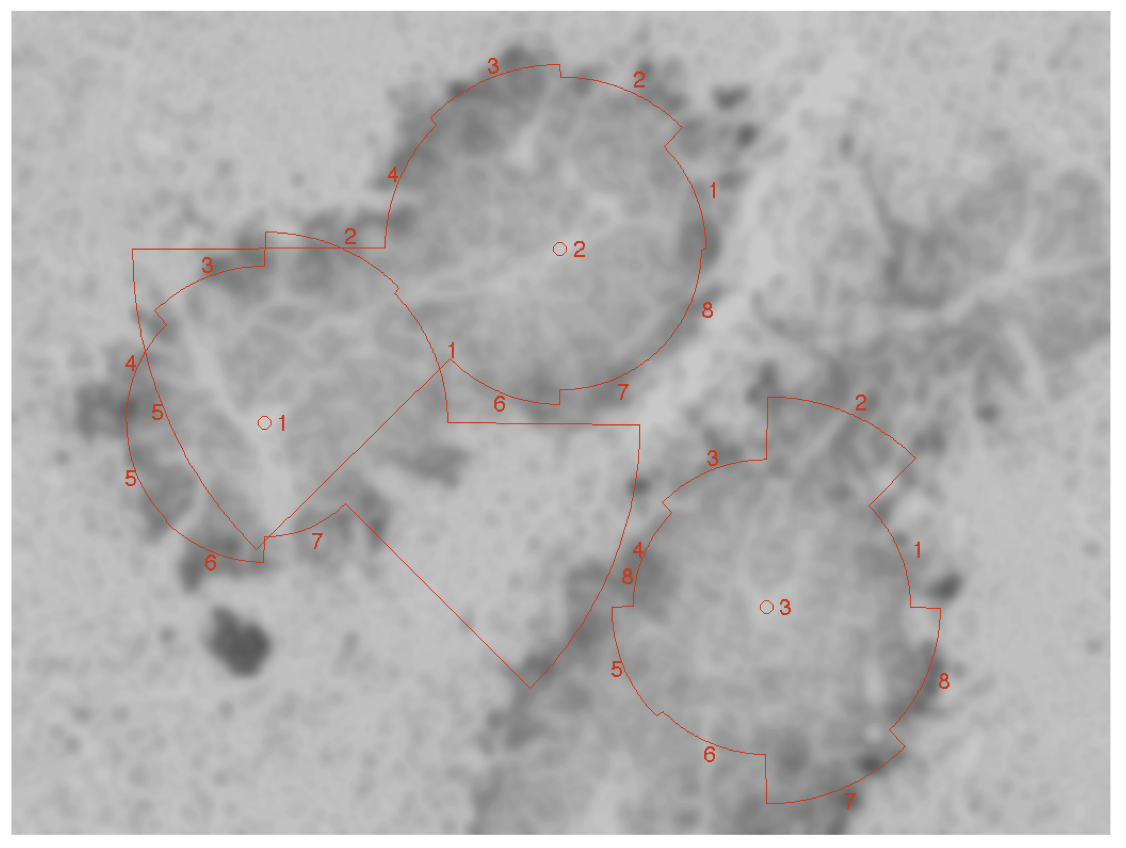

Supplement: S6 Fig — Circle sectors (red) defined by the rm found by our Intensity-Sample-Ray-Bundles algorithm for each bundle of each of the three centers we specified, corresponding to vessel locations in the registered H&E image. Here we show m = 8 sectors (π4 radians per sector) for each center. Sectors are labeled with red numbers, counterclockwise, just outside of the red sector contour. (TIF) [file pone.0153623.s006.tif]

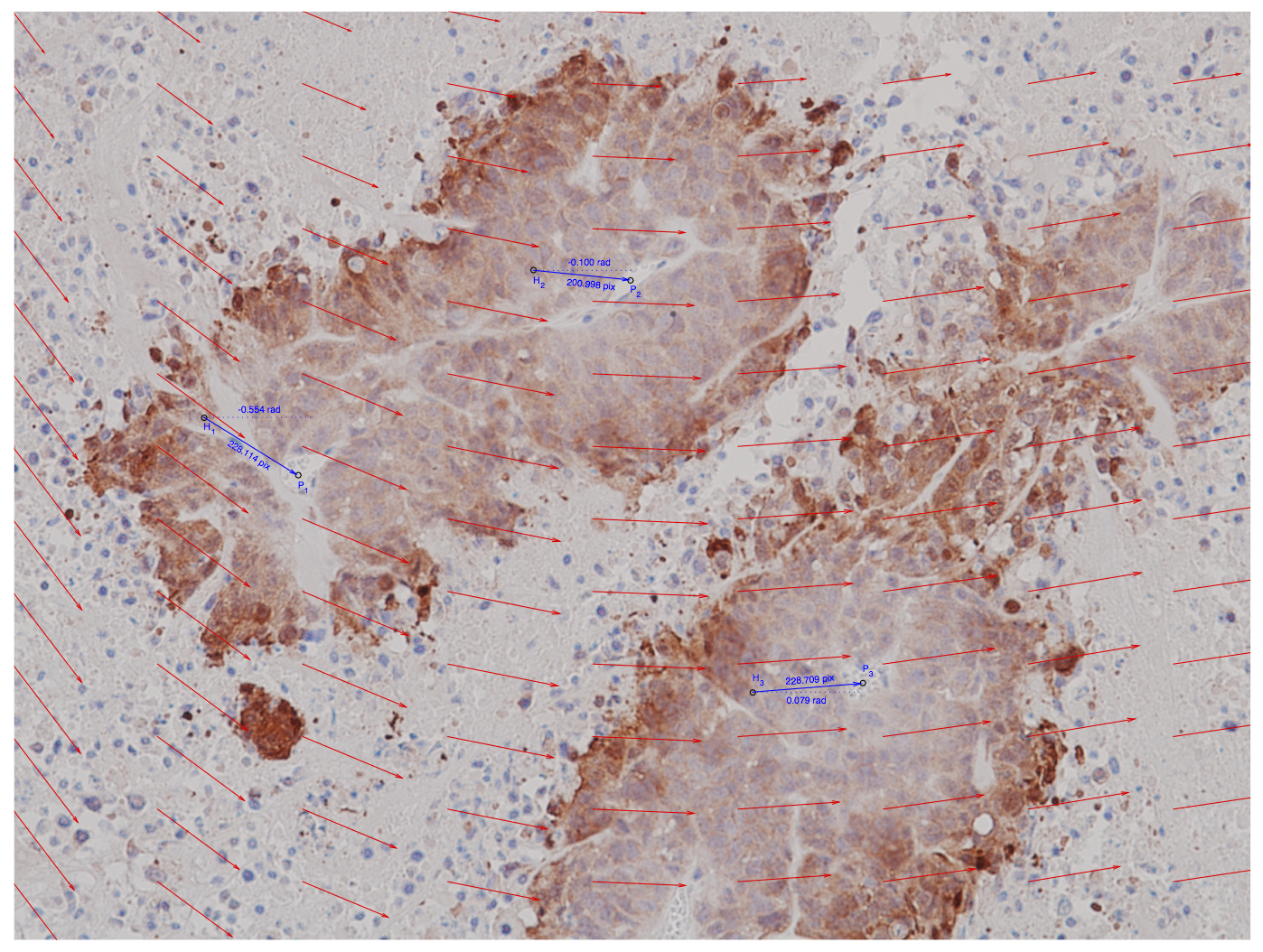

Supplement: S7 Fig — Registering an H&E image (not shown) to a Z-stack-adjacent anti-pimonidazole image (10 μm away). The three blue vectors denote the displacements of the three gradient centers. Each blue vector is labeled with Pi at the head (center position i in the anti-pimonidazole image) and Hi at the tail (the corresponding center position i in the H&E image). The vector lengths (in pixels) are labeled, as are the vector angles (in radians), measured relative to their respective dotted blue horizontal lines. (TIF) [file pone.0153623.s007.tif]

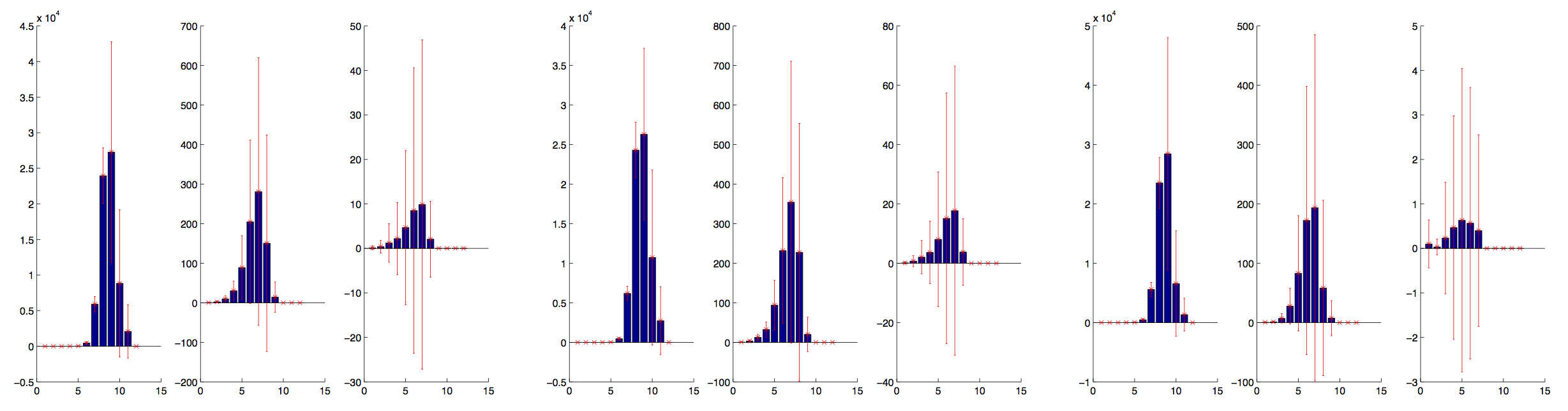

Supplement: S8 Fig — How the Ply-Stats-Quad-Tree algorithm dissects images according to the property of CV in intensity level of a given frame’s pixels: the mean window size profile across the total (left three panels, n = 66), high (middle three panels, n = 36), and low (right three panels, n = 30) sets of images. In each set of three panels, the first, second, and third panels show mean histograms for τ ∈ {0.1, 0.5, 0.9}, respectively. The horizontal axis indicates ply depth, or frame size as computed by x_dim2i×y_dim2i, where i ∈ [0, 12] is the ply depth, and x_dim and y_dim are the x and y dimensions of the whole image, respectively. The vertical axis indicates the mean count of search tree leaves at ply depth i. Error bars show standard deviation. (TIF) [file pone.0153623.s008.tif]

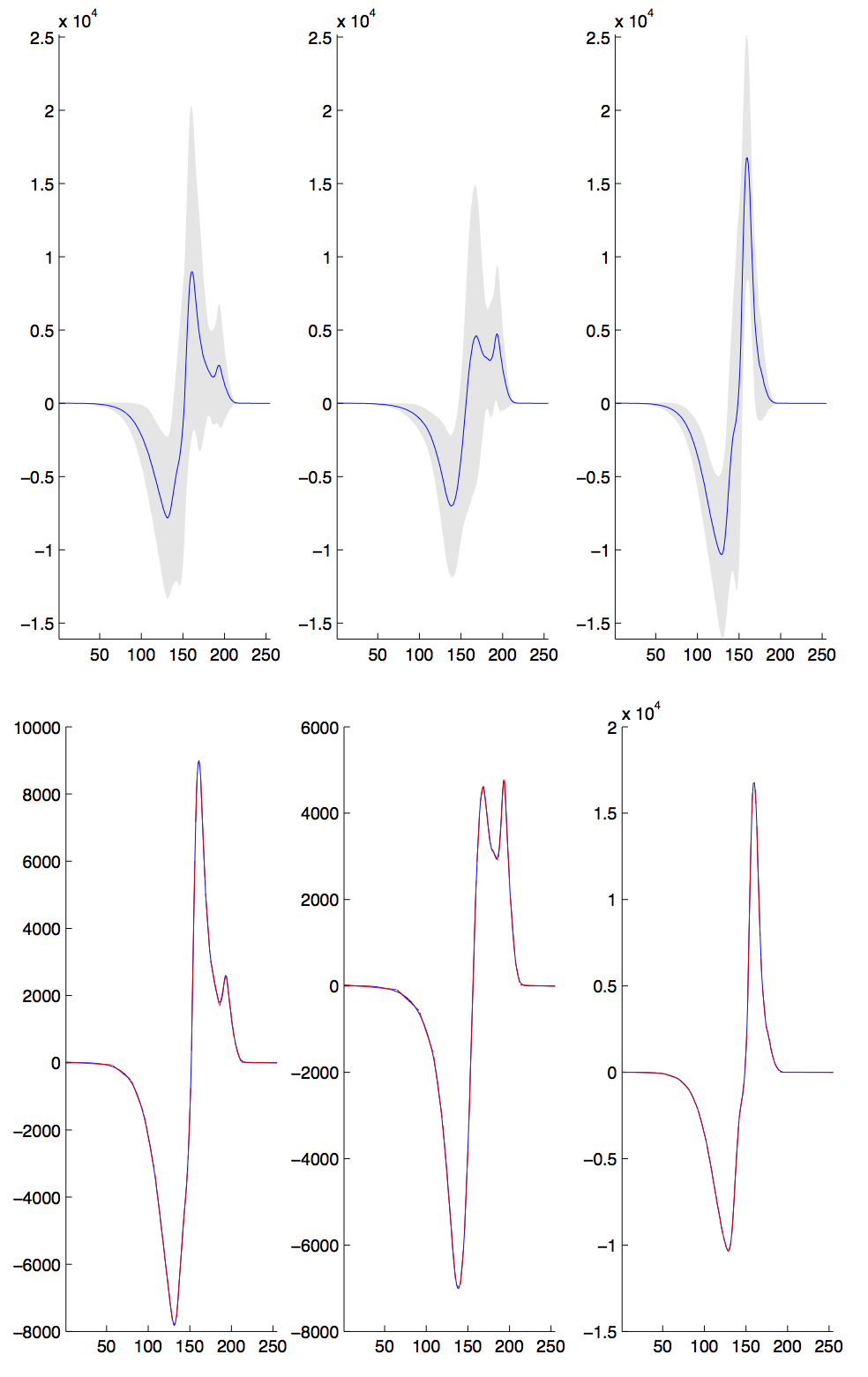

Supplement: S9 Fig — (Top) The mean EPC curve over the total set of images (left, n = 66), the high concentration anti-pimonidazole images (middle, n = 36), and the low concentration anti-pimonidazole images (right, n = 30). Segmented least-squares fits to these curves are given below. The horizontal axis indicates the intensity level threshold τ ∈ [1, 255] applied to the image prior to computing χ. The vertical axis indicates the value of χ computed for each τ. (Bottom) The segmented least-squares fits to the mean EPC curves given above. The horizontal axis indicates the intensity level threshold τ ∈ [1, 255] applied to the image prior to computing χ. The vertical axis indicates the value of χ computed for each τ. (TIF) [file pone.0153623.s009.tif]
